# Surface Adhesion Engineering for Armored Metasurfaces and Beyond

**DOI:** 10.1002/advs.202514000

**Published:** 2025-10-17

**Authors:** Lianwei Chen, Chengjun Zhang, Ahai Zhou, Qingsong Wang, Yao Fang, Jiangning Zhou, Xiong Li, Yinghui Guo, Yizhe Zhao, Mingbo Pu, Xiangang Luo

**Affiliations:** ^1^ State Key Laboratory of Optical Field Manipulation Science and Technology, Institute of Optics and Electronics Chinese Academy of Sciences Chengdu 610209 China; ^2^ Research Center on Vector Optical Fields Institute of Optics and Electronics Chinese Academy of Sciences Chengdu 610209 China; ^3^ College of Materials Science and Opto‐Electronic Technology University of Chinese Academy of Sciences Beijing 100049 China

**Keywords:** Metasurface, micro/nano structures, superhydrophobic, surface adhesion

## Abstract

Conventional metasurfaces, despite their ability to manipulate electromagnetic waves, are limited by environmental vulnerabilities such as scratches, contamination, and temperature fluctuations. An armored metasurface engineered via nanoscale interfacial manipulation based on Persson's model is introduced, achieving exceptional robustness. This design offers dust repellency (94.7%), superhydrophobicity (156.3° water contact angle), high‐temperature tolerance (>1000 °C), anti‐scratch resilience (200 cycles), and mechanical durability. Optically, it delivers broadband antireflection (99.1% transmission) and stable phase control (<5% fluctuation) under turbulent conditions (C_n_^2^ ≥ 2.1 × 10^−12^). Integrated into atmospheric optical systems, it enables reliable spatiotemporal vector light manipulation, paving the way for applications in adverse‐weather light detection and ranging (LiDAR), aerospace optics, and durable photonic wearables.

## Graphical abstract

Targeting primary causes of dysfunctionality in optical systems, an “armored” metasurface is developed based on Persson's model and Hertzian theory, enabling anti‐dust, anti‐scratch, anti‐impact, and anti‐water, heat resistance capabilities, alongside excellent optical antireflection performance.

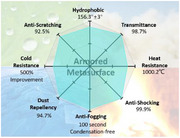

## 1 Introduction

Metasurfaces, comprising 2D arrays of subwavelength nanostructures, have revolutionized optics by providing compact, ultrathin platforms for precise control of electromagnetic waves across spatial, temporal, and polarization domains.^[^ ^1^, ^2^, ^3^, ^4^, ^5^, ^6^ ^]^ However, their widespread adoption in practical systems remains hindered by significant challenges related to scalability, robustness, and environmental adaptability. While traditional metasurfaces excel under controlled conditions, their fragile nanostructures are often susceptible to mechanical degradation, environmental factors such a dust accumulation and ice formation, and optical performance trade‐offs.^[^ ^7^, ^8^, ^9^ ^]^ These vulnerabilities pose significant obstacles for applications requiring reliability, environmental tolerance, and compatibility with high‐power lasers, such as ultralight micro aerial vehicles, where planar optics could significantly reduce payload and energy demands.^[^ ^10^ ^]^ Similarly, large‐aperture systems like the Gran Telescopio Canarias, the world's largest single‐aperture optical telescope, require optical components that remain stable under thermal cycling, wind loading, and contamination. Overcoming these cross‐scale challenges requires a paradigm shift in metasurface design, moving beyond single‐function, delicate optics toward multifunctional, robust photonic interfaces capable of withstanding harsh environments.

Recent efforts in robust surface engineering have demonstrated encouraging progress through the development of abrasion‐resistant, superhydrophobic coatings and micro/nano‐textured interfaces. Strategies include the adhesion layer for bonding strengthening,^[^ ^11^, ^12^ ^]^ abrasion‐bearing discrete microstructures,^[^ ^13^, ^14^, ^15^ ^]^ controlled abrasion by self‐sustaining upper layers,^[^ ^16^, ^17^ ^]^ and dual‐scale micro/nano hybrid structuring.^[^ ^18^ ^]^ These approaches combine nanoscale features for water repellency with microscale structures for mechanical strength. However, such strategies need further improvement to be compatible with metasurface optics, as their exposed nanoscale features, critical for light manipulation, cannot be shielded without sacrificing optical functionality. The central challenge lies in integrating mechanical robustness, environmental adaptability, and advanced optical performance without compromising any aspect.

Here, we introduce a novel class of volumetrically engineered armored metasurfaces designed to overcome the primary causes of optical system dysfunction in extreme physical and optical environments. Leveraging Persson's model and Hertzian theory, we engineered micro‐ and nanostructures to minimize adhesion, creating a versatile platform for particle and environmental control. Through rigorous static and dynamic adhesion analysis of over 12000 particles, we established universal adhesion‐suppression principles that underpin our multifunctional metasurface design. The resulting platform exhibits exceptional durability and environmental resistance, with demonstrated performance including: 94.7% dust repellency, 156.3° water contact angle, high‐temperature tolerance above 1000 °C, anti‐scratch resilience (200 cycles), and mechanical impact tolerance (maintaining optical function after drops and compressive damage, Movie S1, Supporting Information). Optical characterization reveals a high‐transmission, broadband antireflection response (99.1%), coupled with robust phase stability (<5% fluctuation) in turbulent free‐space conditions equivalent to 5 km atmospheric propagation (C_n_^2^ ≥ 2.1 × 10^−12^). This work provides a scalable framework for deploying metasurfaces in demanding applications, from field‐ready light detection and ranging (LiDAR) and space telescopes to wearable photonics and advanced manufacturing, establishing a new paradigm for photonic systems that seamlessly integrate optical sophistication with material resilience.

## 2 Results and Discussion

As shown in Figure 2a, this armored metasurface integrates nine distinct functionalities: dust resistance, water resistance, anti‐condensation, ice resistance, high‐temperature resistance, scratch resistance, impact resistance, enhanced transmittance, and spatial phase modulation (Comparison with other techniques is presented in Table S2, Supporting Information). This manuscript begins with an in‐depth discussion on surface adhesion manipulation, a critical factor in governing the attachment of solid particles to the metasurface. This challenge has long been a bottleneck for conventional metasurfaces, as dust trapped within nanostructures is difficult to remove without causing damage.^[^ ^19^, ^20^, ^21^, ^22^ ^]^ Addressing this issue necessitates a comprehensive examination of surface adhesion physics in the context of metasurfaces, coupled with innovative engineering solutions. Following this, we detail the design, fabrication, and characterization of the armored metasurfaces based on these principles, evaluating the performance across all nine functionalities. Then, the subsequent sections explore two categories of applications: a) metasurfaces for information systems, this application highlights the potential of metasurfaces in long‐range optical systems operating within atmospheric environments, where robustness and environmental adaptability are crucial. b) applications beyond‐metasurface, this segment demonstrates how the principles of surface adhesion manipulation can extend to a broad spectrum of applications, linking metasurface technology to other domains.

### 2.1 Surface Adhesion Manipulation

#### 2.1.1 Governing Equations and Mechanisms

**Figure 1:**
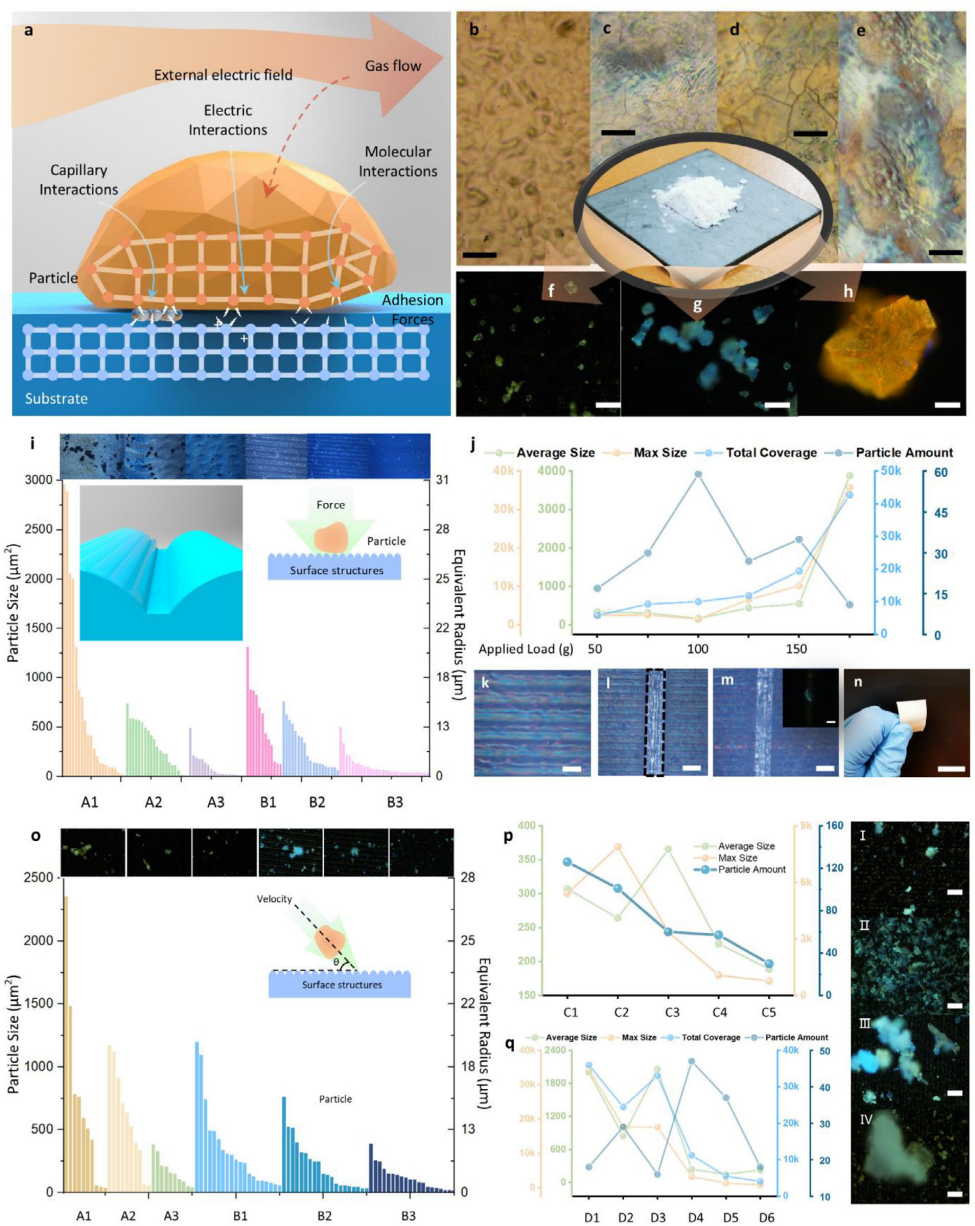
Surface Adhesion and Laser Texturing Analysis. a) Schematic illustrating forces governing surface adhesion. b–e) Microscope images of various laser‐textured metal surfaces, scale bar: 50 µm. f–h) Microscope images of particles remaining on laser‐textured surfaces, scale bar: 50 µm. i) Characterization of particles on different laser‐textured surfaces, with top microscope images showing particles on substrates placed directly in powder. j) Particle retention under varying applied loads. k–m) Selection of particles with specific shapes, scale bars: 50 µm for (e–g). n) Laser texturing of a flexible substrate, scale bar: 1 cm. o) Particle retention on laser‐textured surfaces under kinetic conditions, with top microscope images showing remaining particles. p) Particle detachment from varying drop heights. q) Particle detachment at different angles. (I‐IV) Microscope images of remaining particles for four distinct cases, scale bar: 100 µm.

Surface adhesion in nature is governed by the interactions between a particle and a substrate across their entire contact area. This adhesion can be quantitatively characterized by calculating the energy required to detach a particle from the substrate, with the key contributing factors illustrated in **Figure** **1**a. Multiple forces influence surface adhesion, primarily including capillary, molecular, and electrostatic interactions. For specific material compositions and morphologies, chemical bonding and mechanical interlocking can also play a role. These adhesion forces depend on the intrinsic properties of the particle, the substrate, and the surrounding environment. Real surfaces exhibit roughness across multiple scales, from microns to nanometers. According to Persson's model and Hertzian contact theory, this roughness can be described using the power spectral density (PSD) function:^[^ ^23^ ^]^

(1) $$C\left(q\right)=\langle \left|h{\left(q\right)}^{2}\right|\rangle$$

where *h*(*q*) represents the surface height function in Fourier space, and *q* is the wavevector associated with the roughness. The adhesion energy can be expressed as:

(2) $$W=\gamma \int_{q_{\min}}^{q_{\max}}qdq$$

where *γ* is the work of adhesion per unit area; *q_max_* and *q_min_* correspond to the highest and lowest wavevectors of the surface roughness. This theory suggests that surface adhesion can be effectively manipulated by tuning the roughness wavevectors. To develop a universal approach based on this concept, several challenges must be addressed. Both particles and substrates exhibit diverse properties, with particle sizes ranging from nanometers to hundreds of microns. To systematically explore these scenarios, we introduce a multi‐cycle laser surface texturing method to precisely engineer surface structures. Unlike conventional laser texturing, which typically completes the process in a single step, our method incrementally constructs the desired textures through multiple processing cycles (Figure 1b–e). In each cycle, laser parameters are carefully set just above the ablation threshold, ensuring that only the uppermost thin layer of the substrate is modified. This controlled, layer‐by‐layer material removal enables precise tailoring of surface structures. Moreover, laser parameters can be dynamically adjusted in each cycle to introduce varying texturing effects. Experimental results demonstrate that diverse nanostructures can be fabricated, and by strategically combining processing cycles, we can fine‐tune the surface texture to achieve a broad range of nanoscale patterns, optimizing adhesion control. Next, we experimentally characterize surface adhesion for particles of varying sizes, shapes, and compositions (examples shown in Figure 1f–h). Surface adhesion is analyzed under two conditions: 1) Static Condition: Particles rest on and make contact with the substrate. 2) Dynamic Condition: Particles impact the substrate and either adhere or bounce off.

#### 2.1.2 Static Surface Adhesion

To investigate static adhesion, various textured samples were fabricated, and their adhesion properties were tested under different conditions. The first experiment examines a common contact scenario between a particle and a textured interface (Figure 1i). Stainless steel substrates with different surface textures were placed over a layer of white coffee particles, chosen for their broad size distribution (ranging from 100 nm to hundreds of microns) and irregular shapes. The substrates were then removed and analyzed under an optical microscope to quantify the sizes of adhered particles within an area of 870 × 653 µm (≈568110 µm^2^). The methodology for optical imaging, texture fabrication, and data processing is detailed in Note S1 (Supporting Information).

Two groups of samples were fabricated based on adhesion mechanisms (Figure 1i):

1. **Group A**: Homogeneous nano‐textured surfaces (Samples A1–A3), with decreasing texture sizes. These nano‐textures reduce adhesion due to weaker interaction forces (Equation 1). However, small particles that fit into the grooves exhibit increased adhesion. Experimental results confirm this behavior: the average particle sizes on Samples A1, A2, and A3 decreased from 739 to 328 µm^2^ and 97 µm^2^, respectively. While effective at repelling larger particles, this strategy functions similarly to a sieving process and does not prevent smaller particles from adhering.
2. **Group B**: Hybrid structures combining nano‐textures with micro‐grooves (Samples B1‐B3), where micro‐groove gaps decrease from 50 to 10 µm. The nano‐textures reduce surface adhesion, while the micro‐grooves selectively attract particles of specific sizes. Experimental results show that Sample B2 exhibits the best adhesion control, achieving a narrower particle size distribution.

To examine the effect of external force on adhesion, Sample B2 was pressed onto a particle layer with varying loads (Figure 1j). When the pressing force increased from 0.49 to 0.98N, more particles adhered due to greater contact. However, beyond 0.98 N, particle clustering occurred, reducing selective adhesion. The findings suggest an optimal pressing force threshold: below this value, increased force improves particle separation; but above it, clustering disrupts selection.

Besides size and force, particle shape also affects adhesion. A similar design strategy was applied to selectively capture high‐aspect‐ratio particles (e.g., bar‐shaped particles). First, nano‐textures were fabricated to repel most particles (Figure 1k). A single laser‐ablated groove (Figure 1l) was then introduced to attract particles of matching shape. After pressing the substrate onto white coffee particles and characterizing the adhered particles, results confirmed that elongated particles preferentially adhered (Figure 1m). This flexible fabrication method allows customization with different groove shapes (e.g., triangles, squares) for specific particle morphologies, enabling applications in optical sensing. Additionally, surface texturing on flexible substrates (e.g., aluminum foil, Figure 1n) extends its applicability to environmental monitoring and wearable electronics.

#### 2.1.3 Dynamic Surface Adhesion

The dynamic adhesion experiment investigates the influence of angle of incidence and velocities on particle attachment (Figure 1o). Laser‐textured substrates were positioned on a tilting stage at 45°, and 1 g of white coffee particles was dropped from a height of 5 cm. The number and size distribution of adhered particles were analyzed using optical microscopy. Group A and B samples, fabricated under the same conditions as in static experiments, exhibited similar trends: decreasing texture size repelled larger particles, and Sample B3 achieved the best separation performance with uniform particle sizes. However, the average particle size in dynamic conditions was smaller than in static cases. As particles impact the substrate, those with insufficient adhesion roll off, influenced by the velocity of incidence and surface tilt. Unlike static experiments, clustering due to external force was not a significant factor. To further explore these effects, experiments were conducted with varying incident velocities and tilt angles (Figure 1p,q; Figure S1, Supporting Information):

1. **Effect of Incident Velocity**: Dropping heights ranged from 1 to 8 cm (C1 to C6). Higher velocity results in less attached particles, as particles retain greater kinetic energy, requiring a longer surface travel distance before coming to rest. This reduces clustering and results in fewer, smaller adhered particles.
2. **Effect of Tilting Angle**: With a fixed drop height of 5 cm, tilt angles varied from 10° to 60° (D1 to D6). At angles below 30°, particles accumulated and formed clusters, while the best separation was observed at 40° angle. Beyond 40°, excessive tilt caused even desired‐sized particles to roll off, reducing separation effectiveness.

These observations categorize dynamic adhesion outcomes into four cases:

- Optimized Separation: Particles of desired size remain attached (optimized tilt and velocity).
- Over‐Adhesion: Excessive adhesion leads to unwanted particle retention and clustering (low tilt or velocity).
- Poor Separation: Large clusters dominate, reducing selectivity (near‐flat angle, independent of velocity).
- Under‐Adhesion: Desired particles roll off, reducing capture efficiency (high tilt or velocity).

Both nano‐textured and hybrid structures effectively manipulate surface adhesion in static and dynamic conditions. Hybrid structures demonstrated the most precise control, achieving optimal separation by balancing nano‐scale adhesion reduction and micro‐scale particle trapping. By adjusting external forces, texture dimensions, and substrate inclination, this method enables tunable particle manipulation for applications in filtration, sensing, and biomedical fields. Further discussion on the size of the particle to the repellent performance is described in the Note S2 (Supporting Information).

### 2.2 Multifunctional Armored Metasurfaces

With this knowledge, we can manipulate surface adhesion to create particle‐repellent surfaces, evaluating and optimizing the fabrication parameters. However, this approach appears to be in contrast to traditional metasurfaces, which rely on surface patterning for electromagnetic wave manipulation. To address this, we developed a novel metasurface fabrication method in which the nanoantennas are embedded beneath the surface. The fabrication method employed in this study is a recently proposed technique that emphasizes high robustness by creating metaatoms within a transparent substrate. A detailed description of the fabrication process, along with its advantages and limitations, can be found in our previous work.^[^ ^24^ ^]^ The metasurface utilizes laser‐induced birefringence nanopores within silica glass, with an average nanopore diameter ranging from 35  to 45 nm. This metasurface operates based on geometric phase modulation, enabling us to tune the phase shift from 0 to 2π by adjusting the incident light polarization. (The scanning electron microscope (SEM) image of the metasurface can be found in Figure 3e). We then designed two phase maps for metasurfaces based on the conventional Gerchberg‐Saxton (GS) algorithm. One for a hologram pattern and the second one to generate a vortex vector beam, which is used for the anti‐turbulence application presented later. The details of these metasurfaces are given in Note S3 (Supporting Information), and the detailed fabrication processes of the nanostructures are described in Notes S4 and S5 and Figure S16 (Supporting Information). Adjusting laser parameters allows control over nanopore properties, thereby influencing the optical behavior of the metasurfaces.^[^ ^25^ ^]^

Via this approach, we distinctly separate the functional layers responsible for electromagnetic (EM) wave manipulation and surface adhesion control. To modulate surface adhesion, we employ moth‐eye‐like structures integrated onto a quartz surface using reactive ion etching (RIE). This process yields nanocone and nanopillar arrays, with fabrication details outlined in Note S4 (Supporting Information). These nanostructures substantially reduce the effective contact area, endowing the surface with particle‐repellent properties. Characterization experiments, akin to those depicted in Figure 1i, reveal that particle attachment to the treated surface shows a 94.7% particle repellent compared to an untreated reference sample. This reduction corroborates our earlier theoretical predictions. Optical characterization by the microscope further highlights the particle‐repellent effect: regions featuring moth‐eye‐like structures remain largely free of particles, while surrounding untreated areas display significant particle accumulation.

**Figure 2:**
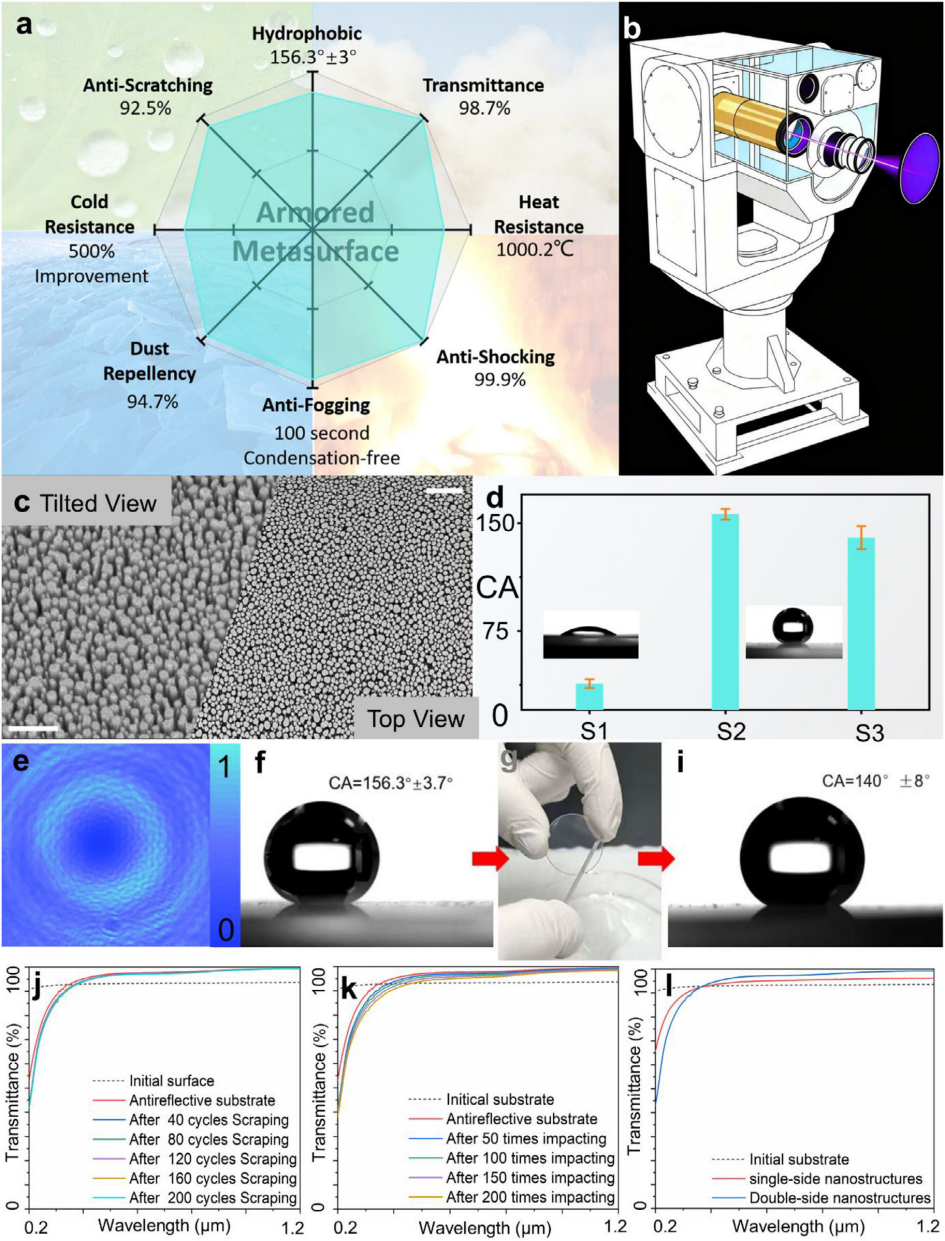
Armored Metasurface Design and Characterization. a) Eight key features of the armored metasurface. b) Armored metasurface integrated into a practical laser communication system. c) SEM images of the armored metasurface, scale bars: 400 nm (tilted view), 1 µm (top view). d) Contact angle measurements of the armored metasurface (S1‐Sample with initial surface; S2‐Sample with superhydrophobic surface; S3‐Sample with superhydrophobic surface after scraping). e) Vortex beam generation by the armored metasurface. f–h) Characterization results before and after scratch testing. i) Transmittance analysis following anti‐scratch testing. j) Transmittance analysis following anti‐impact testing. k) Transmittance enhancement due to surface texturing.

The fabrication of moth‐eye‐inspired nanostructures on a quartz substrate significantly enhances its surface area compared to an untreated planar surface, shifting its inherent hydrophilic nature toward hydrophobicity through a tailored fluorination treatment. Originally hydrophilic due to its high surface energy, the quartz was modified by reducing this energy via vapor deposition of a fluorinated compound or immersion in a fluorosilane‐water solution (1:100 volume ratio), followed by annealing at 80 °C for 2 h to ensure chemical stability of the coating (Figure S6, Supporting Information). This process leverages the nanostructure's topography and the low‐surface‐energy fluorination to dramatically boost water repellency, a critical attribute for advanced optical and functional applications. To quantify this transformation, the treated sample was leveled on a contact angle goniometer (JC2000D, Powereach) stage, and an 8 µL deionized water droplet was dispensed via micro‐syringe. The droplet‐surface interaction was captured using a high‐speed charge coupled device (CCD) camera and analyzed with specialized software (Figure S7, Supporting Information), yielding a contact angle of ≈156.3°, as shown in **Figure** **2**f. This value, exceeding the 150° threshold, confirms the surface's superhydrophobicity, a result of air entrapment by the moth‐eye‐like nanotopography and the low surface energy of fluorinated layer. Such superhydrophobicity unlocks a suite of advantages, such as sustained dryness, self‐cleaning capabilities, and resistance to biofouling, positioning nanostructured quartz as a versatile platform for photonic and engineering innovations.

To rigorously assess the resilience of this metasurface under extreme conditions, we evaluated its performance in both high‐ and low‐temperature environments. High‐temperature exposure typically induces melting in conventional metallic metasurfaces, as their nanostructures, owing to elevated surface area, exhibit lower melting points than their bulk counterparts (e.g., nanostructured gold ≤ 800 °C, silver ≤ 300 °C, copper ≤ 800 °C).^[^ ^26^, ^27^ ^]^ To test high‐temperature stability, we subjected the metasurface to a thermal shock at 1000 °C for 30 min, a regime sufficient to liquefy most nanostructured metals and alter surface morphology. Pre‐ and post‐treatment transmittance characterization revealed that the dimensions of the moth‐eye‐inspired nanostructures remained consistent, with no evidence of melting or structural degradation, which is shown in Figure S8 (Supporting Information). This remarkable thermal endurance likely stems from material composition and design of the metasurface, which defy the limitations of traditional metallic nanostructures. These findings underscore the potential of metasurface for optical applications in extreme terrestrial environments and even near‐space scenarios, where thermal fluctuations are pronounced. We also conducted low‐temperature testing to evaluate the anti‐icing performance of the device surface at −20 °C. This was assessed by comparing the freezing time of water droplets on the surface. Specifically, an 8 µL droplet of deionized water was placed on the device, and its freezing process was recorded in real time using a high‐speed CCD camera. The freezing time was determined as the moment when the droplet's apex transformed into a sharp conical shape. The untreated surface exhibited complete freezing within 18 s, whereas the structured surface significantly delayed freezing, taking up to 90 s, representing a fivefold increase in anti‐icing performance, as shown in Figure S9 (Supporting Information). The details of the condensation testing are found in Figure S10 (Supporting Information). The deionized water was poured into the beaker, and then heated to boiling through the heating table. The metasurfaces without surface chemical treatment and surface hydrophobic treatment were placed above the beaker, respectively. The treated metasurface remains transparent after 100 s, while the water droplets condense on the untreated surface.

Surface mechanical damage is a primary factor in the failure of metasurfaces. Because only a small fraction of the overall area contacts liquids or particles, these limited contact points experience very high localized pressures under mechanical load. This concentrated stress makes the metasurface inherently fragile and highly susceptible to abrasion. Moreover, abrasion not only damages the nanostructured layer but also exposes the underlying metasurface. This exposure can alter the local surface properties, shifting from hydrophobic to hydrophilic, or from repelling particles to attracting them, and may completely compromise the electromagnetic wave manipulation capabilities of conventional metasurfaces. The selected moth‐eye‐inspired nanostructures with high aspect ratio cylindrical pillars can also overcome these challenges (Figure 2c). The flat, smooth top surfaces of the pillars reduce pressure loading, and their dense, periodic arrangement distributes mechanical stress evenly, much like rocks on a cobblestone road enhance durability. Compared to earlier designs, our structure is optimized to provide a 1125% increased contact area due to the dense packing of pillars with flat top surfaces, benchmarked with the previous milestone.^[^ ^18^ ^]^ We verified this concept with an abrasion test. Upon repeated scraping with a steel blade, the microstructure demonstrated excellent resistance to both vertical pressure and shear forces, and the entire system maintained its constrained equilibrium Cassie‐Baxter state (Figure 2d). Following established evaluation protocols,^[^ ^18^ ^]^ the surface was scratched 200 times under a load of 50 MPa. Notably, while conventional metasurfaces in our previous work could barely withstand a single compression, the contact angle of our optimized design remained nearly unchanged (from 156° to 140°, as shown in Figure 2f–h). The transmittance after every 40 cycles of scratching is also tested, which also remains nearly unchanged (Figure 2i). To probe real‐world durability, we conducted a drop‐and‐step test simulating practical handling stresses (Movie S1, Supporting Information). A metasurface engineered to generate a hologram pattern was assembled, dropped from a height of 1 m, and subsequently subjected to a compressive load by a 45.1 kg researcher. Remarkably, the metasurface was reinstalled in the holographic setup post‐test and reproduced the original pattern with negligible deviation. Subsequently, 200 cycles of compressing tests on the metasurface under the same conditions are conducted to characterize the robustness of the surface microstructures and optical properties. The results showed that the surface nanostructures remain intact without obvious damage, and also exhibt excellent antireflective performance (Figure S11, Supporting Information). This resilience highlights the design's potential for applications requiring sustained EM performance under harsh mechanical conditions. To quantitatively study such anti‐impact performance, the surface of the sample to be tested is impacted with a small metal block at an impact speed of 2 cm s^−1^, applying an impact force of ≈795 kPa (10 N impact force, with a direct contact area of 4 mm in diameter). The device transmittance is tested after every 50 repeated impacts. The experimental results, as shown in Figure 2j, indicate that after 200 impacts, the average transmittance of the sample decreases by less than 7%. Nanostructures after surface treatment remain stable with no obvious damage (Figure S12, Supporting Inforamtion). The beam quality variations after 200 cycles of scraping and impacting of vortex light are tested using a beam quality analyzer. The light beam with no obvious distortion indicates that the armor metasurface possesses excellent mechanical stability (Figure S13, Supporting Information).

Optical coatings are essential for manipulating transmission in photonic components, particularly in high‐power applications where reflection mitigation is critical. Uncontrolled reflections can elevate surface temperatures, induce interfacial deformation, and distort wavefronts, risking severe system damage. Conventional metasurfaces, with their nanostructured surfaces, are incompatible with traditional optical coatings, limiting their utility in high‐power laser systems. To address this, our selected moth‐eye‐inspired nanostructures with nanoscale dimensions tailored provide broadband antireflection. The moth‐eye structures, consisting of high‐aspect‐ratio nanocones, are engineered with a characteristic period (Λ) significantly smaller than the incident wavelength (Λ ≤ 0.1λ, for λ = 1064 nm). At these scales, diffraction is negligible, enabling the nanostructure to function as an effective medium with a spatially uniform refractive index. Applying equivalent medium theory, we model the nanocone array on a quartz substrate as a homogeneous thin film with a gradient refractive index, transitioning smoothly from that of quartz (n ≈1.46) to air (*n* = 1). This gradient suppresses Fresnel reflections, conferring exceptional antireflection properties across a broad spectral range. We fabricated the nanocone structures on quartz substrates and characterized their optical performance (Figure 2k; Figure S14, Supporting Information). The fabricated nanostructures on both sides of the quartz substrate exhibit high transmittance increasing from 93.5% (1064 nm, initial substrates) to 99.1% (1064 nm, double‐sided nanostructures). Transmittance measurements reveal a reduction of 1.4% at 1064 nm, with broadband and wide‐angle antireflection sustained over 1000 nm and incidence angles up to 55°. These results, underpinned by the Fresnel reflection framework, outperform conventional metasurfaces lacking such nanostructuring. The enhanced transmission and thermal stability of this design mitigate the risks of wavefront distortion and surface damage under high‐power illumination, as validated in laser‐induced damage threshold tests (Figure S15, Supporting Information). In the 1‐on‐1 testing, the damage threshold exceeds 45 J cm^−2^ (1064 nm, 10 ns). In the R‐on‐1 testing, the damage threshold exceeds 100 J cm^−2^ (1064 nm, 10 ns). This approach eliminates the need for additional coatings, integrating antireflection directly into the metasurface architecture. By enabling compatibility with high‐power laser systems, our armored metasurface expands the scope of nanophotonic applications, from ultrafast optics to laser‐based manufacturing. Multi‐factor influences of the armored metasurfaces in the environment is mentioned in Note S6 (Supporting Information).

### 2.3 Applications

#### 2.3.1 Anti‐Turbulence Information Transmission

We first implemented this metasurface to demonstrate anti‐turbulence optical signal transmission. The armored metasurface effectively mitigates environmental challenges such as extreme temperatures, dust, and raindrops, making it highly suitable for outdoor applications. As Richard P. Feynman noted, turbulence remains one of the most significant unsolved problems in classical physics. Atmospheric turbulence, in particular, disrupts optical wave propagation due to the non‐uniform refractive index distribution, leading to severe wavefront aberrations. These spatially varying distortions degrade the signal‐to‐noise ratio (SNR) and spatial resolution of optical systems, posing a fundamental challenge in free‐space optical communications, astronomical imaging, and remote sensing.

**Figure 3:**
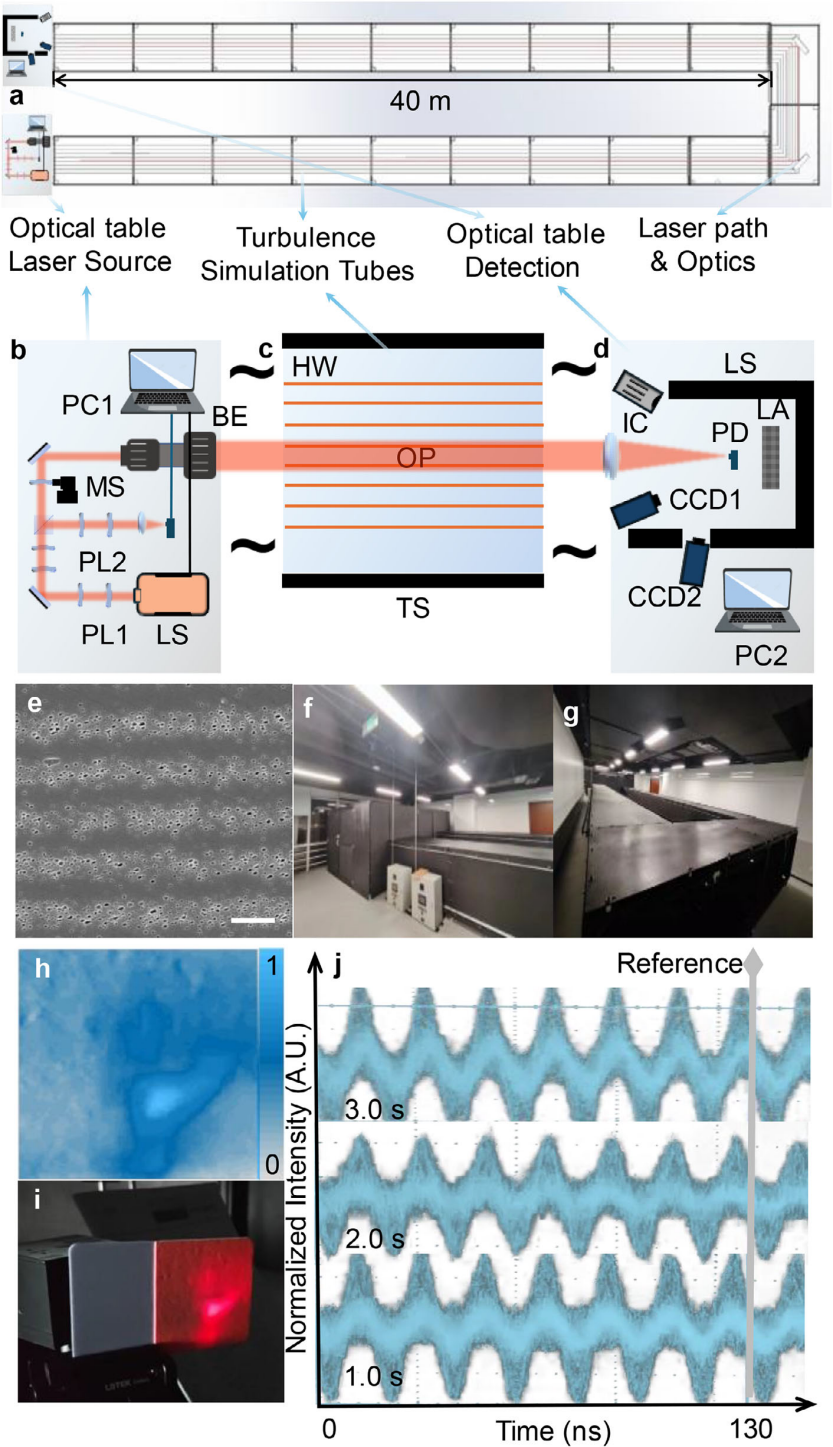
Anti‐Turbulence Application Experiments and Results. a) Schematic of the turbulence simulator experimental setup with two 40 m arms. b–d) Components of the turbulence test system: laser source and modulators, turbulence generator, and characterization setup. e) SEM image of nanostructures in the armored metasurface, scale bar: 1 µm. f,g) Optical images of the anti‐turbulence characterization facility. h) Intensity profile of the laser spot transmitted through turbulence. i) Optical image of the laser spot captured using a laser viewing card. j) Phase stability test under the turbulence conditions with fluctuating intensity.

To address this, we utilized the vortex vector beam generated by the metasurface to produce a vortex laser beam (Figure 2e), which has extensive practical applications in outdoor environments (Figure 2b). The experimental setup is illustrated in **Figure** **3**a. This beam was employed in anti‐turbulence testing, where the metasurface modulates a synthetic wave by leveraging the principle that coherent light at two closely spaced wavelengths (λ_1_, λ_2_) traveling through a scattering medium retains phase information at scales exceeding a synthetic wavelength (SWL), defined as Λ ≫ λ_1_, λ_2_. The mathematical framework underpinning this concept enables phase distortion correction caused by turbulence. The synthetic phase, expressed as ∠E(Λ) = ϕ(Λ) = ϕ(λ_1_) – ϕ(λ_2_), is inherently robust against turbulence‐induced degradation.

To validate this approach, we constructed an 80 m turbulence simulation setup (Figure 3b–d) to replicate varying turbulence conditions. This controlled facility provided an indoor shielded tunnel with minimal ambient disturbance, allowing precise experimental testing (Figure 3f,g). The turbulence simulation was designed to mimic natural atmospheric turbulence, which arises from solar‐surface heating and convective mixing. This effect was emulated using long electrical heating wires suspended below the laser path to heat the air and induce mixing. Unlike static phasefront distortions generated by phase plates, this method produces a well‐characterized statistical distribution of eddies consistent with naturally occurring turbulence.

The turbulence generator consisted of eight independent zones spanning 80 m. Each zone contained four pairs of heating wires, uniformly distributed across a 10 cm width, parallel to the laser propagation direction. Each zone was independently controlled via a computer system, with integrated thermocouples to monitor temperature. While the present experiments maintained a uniform setpoint across all zones, the system allows for individualized control to generate custom longitudinal turbulence profiles. The laser beam was positioned 70 cm above the heating elements, ensuring sufficient propagation distance for the turbulence to develop into a von Kármán‐type spectrum.

The laser source was positioned at the entrance of the turbulence simulator, where the modulated synthetic wave propagated through the turbulent medium before reaching the detection system at the exit. The transmitted wave was captured and analyzed using a CCD camera and an oscilloscope. The results, shown in Figure 3h–j, along with the accompanying video (Movie S2, Supporting Information), demonstrate that even under significant turbulence, the phase remains remarkably stable. During testing, the heating wires were set at 90 °C, generating turbulence with an atmospheric structure constant of refractive index (C_n_^2^) measured at 2.16 × 10^−12^. This condition is equivalent to the turbulence typically observed in the middle of the night over a 5 km optical path. As shown in Figure 3h,i, the beam profile undergoes severe distortion at the exit, as captured by the CCD image of the laser‐illuminated fluorescent screen. The intensity of the vortex beam, recorded by the detector, fluctuates significantly—ranging from 0 to over 1.6 times the intensity measured under turbulence‐free conditions. Despite these dramatic intensity variations, the phase profile remains highly robust. As demonstrated in Figure 3j, the periodicity of each cycle is maintained at 12.5 ns, with phase fluctuations remaining below 5% throughout the captured duration. Notably, intensity fluctuations have no impact on phase stability, confirming the turbulence resistance of the synthetic vortex vector beam.

#### 2.3.2 Applications Beyond Metasurface Platforms

Furthermore, such a surface engineering technique is not confined to metasurface applications; its versatility extends to particle manipulation across diverse scenarios. At the end, we briefly explore the potential of tailored surface textures to enable both dynamic and static manipulation of particles, demonstrating scalable, efficient, and selective systems with broad applicability:

#### 2.3.3 Dynamic Particle Manipulation: Rotational Separator Design

**Figure 4:**
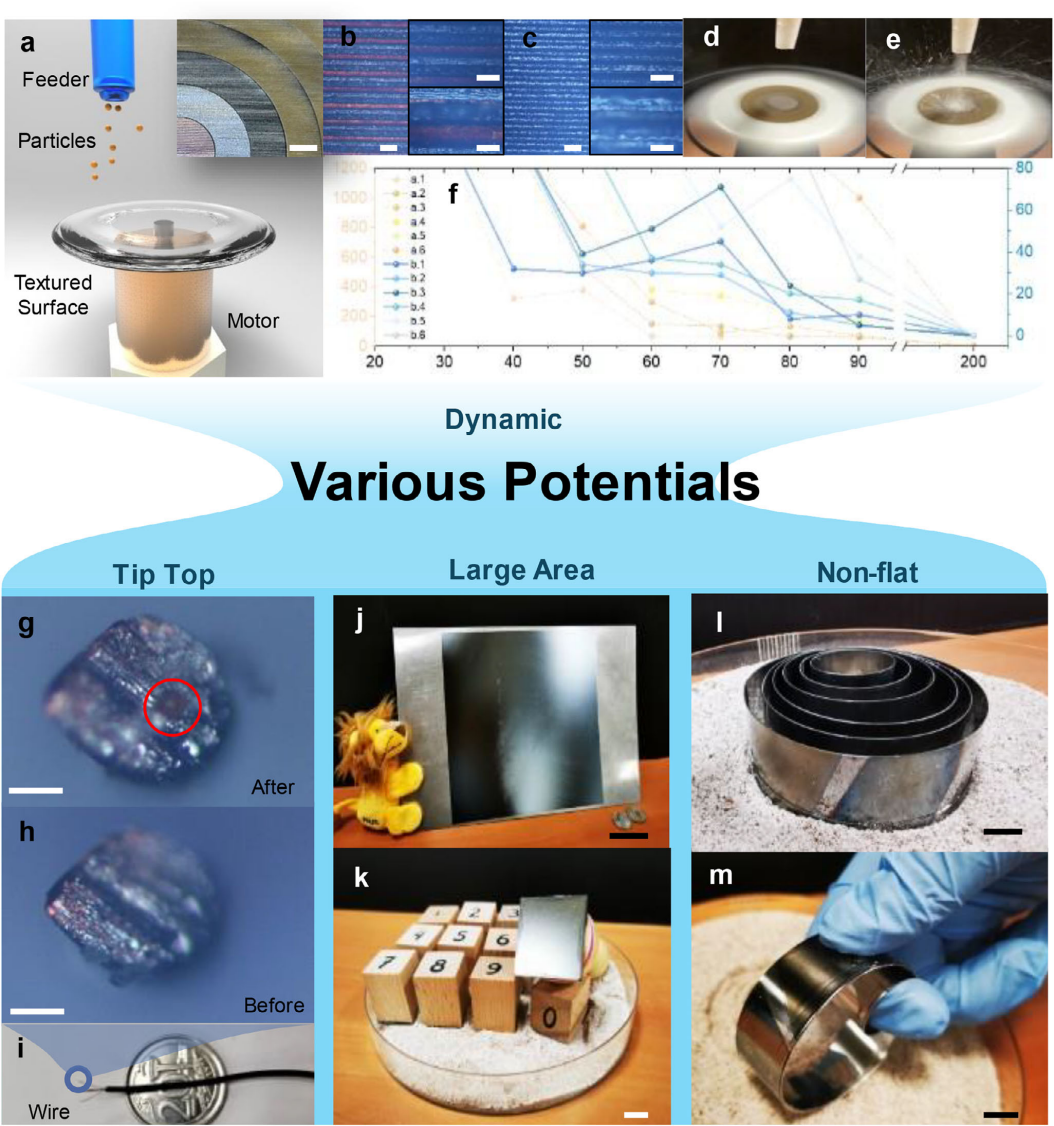
Non‐Optical Applications of Surface Texturing. a) Schematic of the rolling disk particle selector, with top‐right optical image of the textured surface, scale bar: 2 mm. b,c) Microscope images of the textured surface from the disk's center to its outer edge, scale bars: 100 µm (left and top‐right), 40 µm (bottom‐right). d,e) Optical images of the particle selection process. f) Remaining particles in different regions of the rotating disk at varying rotation frequencies. g,h) Microscope images of the textured wire tip with and without selected particles, scale bar: 50 µm. i) Optical image of the processed wire. j) Large‐area laser‐processed metal substrate, scale bar: 5 cm. k) Modular particle selection stamps, scale bar: 1 cm. l,m) Surface‐textured particle roller and its functionality, scale bar: 1 cm.

We first showcase a dynamic particle separation system leveraging centrifugal forces and surface texture variations. A rotational separator was engineered, comprising a textured plate mounted on a precision motor (**Figure** **4**a). The plate's surface consists of concentric rings, each laser‐textured with distinct parameters, texture size decreasing radially from the innermost to the outermost ring (Figure 4b,c). A particle feeder dispenses 2 g of white coffee particles onto the rotating plate, driven at a constant angular velocity (Figure 4d). Upon contact, particles with specific morphologies are selectively captured by the textured regions, while others are ejected by centrifugal forces (Figure 4e). This separation process completes within seconds, offering near‐instantaneous selectivity.

To evaluate performance, we characterized the retained particles across the rings as a function of rotational frequency (Figure 4f). At frequencies below 40 Hz, insufficient centrifugal force results in minimal particle detachment. As frequency increases, outer‐ring particles are preferentially ejected, reflecting the radial dependence of centrifugal force. An optimal frequency of ≈70 Hz balances adhesion and centrifugal forces, retaining only particles with desired shapes while ejecting others with lower surface affinity. Beyond this threshold, particle retention decreases monotonically, with near‐complete ejection observed at 200 Hz. This system demonstrates rapid, size‐selective separation and holds promise for continuous operation, for example, by integrating a brush mechanism to collect retained particles, highlighting its potential in industrial sorting applications.

#### 2.3.4 Static Particle Manipulation: Multifaceted Applications

Beyond dynamic systems, we demonstrate three static applications of laser‐textured surfaces, each addressing distinct challenges in precision and scale. A brief summary of commercial and pre‐commercial applications is provided in Table S3 (Supporting Information).

  - **Tip‐Top Manipulation**: For microscale manipulation, we developed a laser texturing setup to functionalize wire tips. Precise alignment of the cross‐section of the tip with the laser focal spot is critical, particularly for soft or thin materials prone to displacement by ablation‐induced shockwaves (Figure 4i). To overcome this, we pre‐fabricated guide holes in a paper substrate, threading the wire tip through and aligning it coplanar with the paper. The tip's position was mapped, and laser texturing was performed by focusing on this plane. The resulting textured tip successfully captured individual white coffee particles (Figure 4g,h), underscoring its potential for single‐particle handling in microengineering or biological applications.
  - **Large‐Area Texturing**: Scaling up to macroscopic areas, we employed a robotic arm equipped with a fiber laser to texture a 20 × 20 cm stainless‐steel substrate (Figure 4j). Uniformity was ensured by an initial outline‐texturing cycle, verifying consistent texture depth across the substrate. Particle separation tests at the four corners confirmed homogeneous performance, enabling the fabrication of ten distinct “stamps” with varied textures (Figure 4k). These stamps act as rapid particle analyzers: pressing them onto a sample selectively captures particles based on texture‐specific adhesion, offering immediate, large‐area detection of particle properties, a valuable tool for quality control or environmental monitoring.
  - **Non‐Planar Surfaces**: Texturing non‐flat objects requires dynamic focal adjustment to maintain uniformity along curved surfaces. Using a galvo‐scanner with an extended working distance and a rotational wheel setup, we textured stainless‐steel wheels of varying diameters (Figure 4l). Each wheel's surface was segmented into six regions, each with unique texture parameters. Rolling the wheel over particles resulted in selective capture by different segments (Figure 4m), demonstrating morphology‐dependent adhesion. Such capabilities are industrially relevant, e.g., capturing dust in engine exhausts to enhance longevity and reduce emissions—illustrating the adaptability of this approach to complex geometries.

#### 2.3.5 Compatibility of the Conventional Techniques

Recognizing the critical need for metasurface nanofabrication techniques to align with standard semiconductor processes for scalability and cost‐effectiveness, our team has been actively pursuing complementary metal oxide semiconductor (CMOS)‐compatible methods, as evidenced by our recent work.^[^ ^28^, ^29^ ^]^ The method here is a single‐step patterning approach using femtosecond lasers to create birefringent structures in quartz, enabling efficient light‐field modulation with high laser damage threshold, excellent mechanical stability, and superior conversion efficiency. While this method differs from conventional multi‐step processes like lithography and etching, it offers simplicity and potential compatibility with CMOS workflows, as subsequent steps such as etching or coating can be integrated if needed. Although further studies are planned to fully align this technique with diverse metasurface fabrication methods, its promising attributes suggest significant potential for advanced optical applications.

## 3 Conclusion

In this work, we have demonstrated an innovative armored metasurface platform that achieves robust optical performance while enhancing mechanical durability and environmental resilience. This metasurface retains high efficiency and tunability despite external perturbations, marking a significant advancement in practical metasurface applications. The experimental and theoretical analyses confirm that our approach successfully mitigates common challenges such as structural degradation, thermal instability, and susceptibility to mechanical stress. The versatility of this armored metasurface opens new possibilities for applications in harsh environments, including aerospace, biomedical sensing, and industrial optical systems. Furthermore, our findings suggest potential avenues for integrating active tuning mechanisms and multi‐functional capabilities, further expanding the scope of metasurface‐based technologies. By addressing the longstanding issue of metasurface fragility, this study paves the way for real‐world implementations of high‐performance, resilient metasurfaces in next‐generation optical and photonic systems.

## 4 Experimental Section

### Fabrication of the Antireflective Nanostructures

The fabrication of antireflective nanostructures mainly involves the following steps: 1) Depositing a metal thin film (Au) on the quartz surface; 2) Converting the gold film into gold nanoparticles under a high‐temperature environment; 3) Fabricating high‐aspect‐ratio nanopillar structures on the quartz surface via reactive ion etching (RIE). Subsequently, a fluorination treatment was applied to the surface of the nanopillar structures to obtain a surface anti‐reflective structure with superhydrophobic properties. The more detailed information can be found in Note S4 (Supporting Information).

### Laser‐Based Fabrication Process for the Birefringent Nanostructures

The construction of birefringent nanostructures is mainly achieved by means of laser direct writing inside quartz. The designed metasurface was fabricated through line‐by‐line scanning and layer‐by‐layer scanning. The detailed fabrication process can be found in Note S5 (Supporting Information).

## Conflict of Interest

The authors declare no conflict of interest.

## Supporting information



Supporting Information

Supplemental Movie 1

Supplemental Movie 2

## Data Availability

Research data are not shared.

## References

[1] N. A. Rubin , Z. Shi , F. Capasso , Adv. Opt. Photonics. 2022, 13, 836.

[2] N. Yu , P. Genevet , M. A. Kats , F. Aieta , J.‐P. Tetienne , F. Capasso , Z. Gaburro , Science 2011, 334, 333. 21885733 10.1126/science.1210713

[3] H. Gao , X. Fan , Y. Wang , Y. Liu , X. Wang , K. Xu , L. Deng , C. Zeng , T. Li , J. Xia , W. Xiong , Opto‐Electron Sci. 2023, 2, 220026.

[4] Z. Wang , W. Pan , Y. He , Z. Zhu , X. Jin , M. Liu , S. Ma , Q. He , S. Sun , L. Zhou , Opto‐Electron Sci. 2025, 4, 240024.

[5] X. Xie , M. Pu , J. Jin , M. Xu , Y. Guo , X. Li , P. Gao , X. Ma , X. Luo , Phys. Rev. Lett. 2021, 126, 183902. 34018802 10.1103/PhysRevLett.126.183902

[6] M. Pu , X. Li , X. Ma , Y. Wang , Z. Zhao , C. Wang , C. Hu , P. Gao , C. Huang , H. Ren , X. Li , F. Qin , J. Yang , M. Gu , M. Hong , X. Luo , Sci. Adv. 2015, 1, 1500396. 10.1126/sciadv.1500396 PMC4646797 26601283

[7] A. H. Dorrah , F. Capasso , Science 2022, 376, abi6860. 10.1126/science.abi6860 35446661

[8] M. Piccardo , V. Ginis , A. Forbes , S. Mahler , A. A. Friesem , N. Davidson , H. Ren , A. H. Dorrah , F. Capasso , F. T. Dullo , B. S. Ahluwalia , A. Ambrosio , S. Gigan , N. Treps , M. Hiekkamäki , R. Fickler , M. Kues , D. Moss , R. Morandotti , J. Riemensberger , T. J. Kippenberg , J. Faist , G. Scalari , N. Picqué , T. W. Hänsch , G. Cerullo , C. Manzoni , L. A. Lugiato , M. Brambilla , L. Columbo , et al., J. Opt. 2021, 24, 013001.

[9] M. Khorasaninejad , W. T. Chen , R. C. Devlin , J. Oh , A. Y. Zhu , F. Capasso , Science 2016, 352, 1190. 27257251 10.1126/science.aaf6644

[10] W. Shen , J. Peng , R. Ma , J. Wu , J. Li , Z. Liu , J. Leng , X. Yan , M. Qi , Nature 2024, 631, 5374. 10.1038/s41586-024-07609-4 39020037

[11] B. Bhushan , M. Nosonovsky , Acta Mater. 2003, 51, 4331.

[12] W. Zhang , T. Xiang , F. Liu , M. Zhang , W. Gan , X. Zhai , X. Di , Y. Wang , G. Liu , C. Wang , ACS Appl. Mater. Interfaces. 2017, 9, 15776. 28426200 10.1021/acsami.7b02158

[13] V. Kondrashov , J. Rühe , Langmuir 2014, 30, 4342. 24628022 10.1021/la500395e

[14] G. Azimi , R. Dhiman , H.‐M. Kwon , A. T. Paxson , K. K. Varanasi , Nat. Mater. 2013, 12, 315. 23333998 10.1038/nmat3545

[15] R. Han , Y. Zhang , Q. Jiang , L. Chen , K. Cao , S. Zhang , D. Feng , Z. Sun , T. Jia , Opto‐Electron Sci. 2024, 3, 230013.

[16] C. Peng , Z. Chen , M. K. Tiwari , Nat. Mater. 2018, 17, 355. 29581573 10.1038/s41563-018-0044-2

[17] X. Deng , L. Mammen , H.‐J. Butt , D. Vollmer , Science 2012, 335, 67. 22144464 10.1126/science.1207115

[18] D. Wang , Q. Sun , M. J. Hokkanen , C. Zhang , F.‐Y. Lin , Q. Liu , S.‐P. Zhu , T. Zhou , Q. Chang , B. He , Q. Zhou , L. Chen , Z. Wang , R. H. A. Ras , X. Deng , Nature 2020, 582, 55. 32494077 10.1038/s41586-020-2331-8

[19] K. Zhao , Y. Ha , Y. Guo , M. Pu , Y. Fan , X. Li , F. Zou , L. Wang , S. She , X. Luo , Adv. Opt. Mater. 2024, 12, 2303009.

[20] W. Liu , Z. Li , M. A. Ansari , H. Cheng , J. Tian , X. Chen , S. Chen , Adv. Mater. 2023, 35, 2208884. 10.1002/adma.202208884 37055931

[21] T. Xie , F. Zhang , M. Pu , H. Bao , J. Jin , J. Cai , L. Chen , Y. Guo , X. Feng , Q. He , X. Ma , X. Li , B. Jiang , X. Luo , Laser Photonics Rev. 2023, 17, 2300119.

[22] L. Chen , Y. Li , M. Hong , Adv. Opt. Mater. 2018, 7, 1801130.

[23] B. N. J. Persson , J. Chem. Phys. 2001, 115, 3840.

[24] Q. Wang , Y. Fang , Y. Meng , H. Hao , X. Li , M. Pu , X. Ma , X. Luo , Opto‐Electron. Adv. 2024, 7, 240112.

[25] H. Hao , Y. Fang , Z. Diao , X. Li , L. Chen , Q. Wang , X. Ma , Y. Wang , X. Luo , Photonics Res. 2025, 13, 2487.

[26] S. Inasawa , M. Sugiyama , Y. Yamaguchi , J. Phys. Chem. B 2005, 109, 3104. 16851329 10.1021/jp045167j

[27] L. Chen , Q. Wang , L. Xiong , J. Nanopart. Res. 2017, 19, 300.

[28] T. Kang , T. Zhang , F. Zhang , M. Pu , L. Chen , H. Bao , S. Chen , A. Du , L.i Long , Y. Guo , M. Xu , X. Luo , Adv. Funct. Mater. 2025, 35, 2504593.

[29] F. Zhang , Y. Guo , M. Pu , L. Chen , M. Xu , M. Liao , L. Li , X. Li , X. Ma , X. Luo , Nat. Commun. 2023, 14, 1946. 37029133 10.1038/s41467-023-37510-z PMC10081998

