# Supplementary material for: Surface Adhesion Engineering for Armored Metasurfaces and Beyond
Source: Adv Sci (Weinh). 2025 Oct 17;13(1):e14000. doi: 10.1002/advs.202514000 (PMC12767098; doi:10.1002/advs.202514000)

Supporting Information:

Surface Adhesion Engineering for Armored Metasurfaces and Beyond

Lianwei Chen,† Chengjun Zhang,† Ahai Zhou,† Qingsong Wang, Yao Fang, Jiangning Zhou, Xiong Li,* Yinghui Guo, Yizhe Zhao, Mingbo Pu, Xiangang Luo *

L. Chen, C. Zhang, A. Zhou, Q. Wang, Y. Fang, J. Zhou, X. Li, Y. Guo, Y. Zhao, M. Pu, X. Luo

State Key Laboratory of Optical Field Manipulation Science and Technology, Chinese Academy of Sciences, Chengdu, 610209, China

Email: lixiong@ioe.ac.cn, lxg@ioe.ac.cn

L. Chen, A. Zhou, J. Zhou, Y. Guo, M. Pu, X. Luo

Research Center on Vector Optical Fields, Institute of Optics and Electronics, Chinese Academy of Sciences, Chengdu 610209, China

L. Chen, A. Zhou, X. Li, M. Pu, X. Luo

College of Materials Science and Opto-Electronic Technology, University of Chinese Academy of Sciences, Beijing 100049, China

**Note S1.** The methodology for optical imaging, texture fabrication, and data processing.

**1.1 Optical Imaging and Data Processing (Associated with Figure S1)**

Surface morphologies and residual powder distribution on samples are analyzed using a customized Nikon Eclipse Ni-E upright microscope equipped with both laser and white light illumination sources for enhanced imaging versatility. For powder adhesion measurements, experiments are conducted under controlled conditions at 50% relative humidity to ensure reproducibility. The sample is suspended above a flat powder pile using a precision mounting system. A motorized Y-axis stage lowers the sample to gently contact the powder pile, with contact pressure measured by a high-sensitivity stress sensor positioned beneath the powder bed. This setup ensures accurate quantification of the force applied during adhesion tests.

Post-experiment, the optical microscope is used to characterize the distribution and morphology of residual powder particles on the sample surface. Since the sample surface area exceeds the field of view of the microscope’s objective lens, images are captured at five representative sampling points: the four corners and the geometric center of the sample. These images provide a comprehensive view of powder distribution across the surface. The acquired images are processed using ImageJ, an open-source image analysis software, to quantify the size distribution and spatial arrangement of residual powder particles. To enhance statistical reliability, each experiment is repeated multiple times, and the average values of powder distribution metrics are calculated and reported. The processed data provide insights into adhesion behavior under the tested conditions, supporting further analysis of surface-powder interactions.

**1.2 Texture Fabrication for Stainless Steel**

The base material for this study is commercially available AISI 316L stainless steel, supplied in 1-mm-thick sheets. Prior to laser processing, samples are prepared by cutting the stainless steel into squares using a precision cutting tool. The surfaces are then mechanically polished with 800-grit abrasive paper to achieve a smooth, uniform finish, followed by ultrasonic cleaning in deionized water for 10 minutes to remove surface oxides, contaminants, and polishing residues. This preprocessing ensures a clean and consistent starting surface for laser ablation.

Surface texturing is performed using a nanosecond pulsed fiber laser (YLPN series, IPG) operating at a wavelength of 1064 nm, with a pulse duration of 100 ns, maximum output power of 500 W, pulse energy up to 50 mJ, and a repetition rate adjustable up to 50 kHz. A variable attenuator allows precise tuning of the laser power to suit different texturing requirements. The laser beam is directed using a 3D galvanometric scanner, which provides accurate control over beam movement along the X, Y, and Z axes. For enhanced flexibility in processing complex or non-planar geometries, the scanner is mounted on a Fanuc M20-iB robotic arm, offering additional degrees of freedom. The laser is focused through an f-theta lens, producing a focal spot diameter of approximately 12 µm on the sample surface, ensuring high-resolution texturing.

Programmable patterns, including microgrooves and periodic submicron- to nanoscale fold structures, are generated by varying the laser power (1–400 W), scan spacing (15 µm), and scanning speed (up to 100 mm/s). The laser power is adjusted based on the desired texture and sample properties. Multiple laser scans are performed to create progressive variations in surface nanostructures, as illustrated in Figure 1b–e. With each additional scan, the surface evolves, forming intricate hierarchical structures that enhance the functional properties of the material, such as wettability or adhesion characteristics. The combination of precise laser parameters and robotic control enables the fabrication of tailored surface textures for specific applications.

**Note S2.** Further discussion on the size of the particle to the repellent performance

This study, partially funded by a food industry leader, utilized white coffee particles (100 nm to hundreds of microns) to develop dust-repellent metasurface lenses for optical sensors in white powder production, but to address broader applicability, we compiled Table R1 comparing these to environmental particles like dust (1 nm–100 µm), sand (62.5 µm–2 mm), mud (<2 µm–63 µm), and snow (0.5 mm–cm scale) [US EPA; Hydrologic Engineering Center; Edzwald & O’Melia, 1975; Hobbs, 1974]. While our metasurface effectively repels particles similar in size to white coffee powder, we recognize the need for a comprehensive statistical investigation across diverse particle types and metasurface designs (tailored to target wavelengths and surface dimensions) to establish universal anti-dust performance, with plans to collaborate on a metasurface-particle interaction database. For nanoscale particles (e.g., 50 nm), we calculated adhesion contact areas, 157 nm² on flat surfaces, ~471 nm² between 50 nm-spaced nanowalls, and ~63 nm² on 20 nm nanoholes, showing that dust-repellent properties diminish when particles are smaller than metasurface features.

**Table S1.** Summary of common environmental particles, including material, typical size range, and sources

| Material | Typical Size Range | Notes | Sources |
| --- | --- | --- | --- |
| Dust (airborne) | ~0.001 µm – 100 µm | Includes atmospheric dust, PM10 (<10 µm), PM2.5 (<2.5 µm); household dust often 0.5–100 µm | US Environment Protection Agency |
| Sand | 0.0625 mm – 2 mm (62.5 µm – 2000 µm) | Very fine sand: 62.5–125 µm; Fine: 125–250 µm; Medium: 250–500 µm; Coarse: 500–1000 µm; Very coarse: 1–2 mm | Hydrologic Engineering Center, US Army |
| Mud (silt & clay mix) | Clay: <2 µm; Silt: 2–63 µm | Mud consists of clay + silt; often flocculates into aggregates <20–30 µm | Edzwald JK & O'Melia CR (1975) Clay Distribution in Recent Estuarine Sediments. Clay and Clay minerals, Vol 23 p 39-44 |
| Snow (flakes / crystals) | ~0.5 mm – a few mm typical; can aggregate up to cm scale | Individual crystals are often sub-mm to mm; aggregates can reach several cm; extreme historical claim: 381 mm (15 in) | Hobbs, P.V. 1974. Ice Physics. Oxford: Clarendon Press |

**Note S3.** Specs of the metasurfaces.

**3.1 Metasurface Specs of the Vortex Beam Generator**

Phase map calculation: phase map of classic spiral phase plate

Material: quartz

Period: 1μm

Thickness: 1 mm (25 layers stacking)

Size: 2 mm × 2 mm

Order of phase modulation: continuous

Phase modulation range: 0-2π

**3.2 Metasurface Specs of the Hologram Pattern**

Phase map calculation: conventional GS method

Material: quartz

Period: 3μm

Thickness: 1 mm (25 layers stacking)

Size: 4 mm × 4 mm

Order of phase modulation: continuous

Phase modulation range: 0-2π

**NoteS4.** Fabrication process of the functional nanostructures of armored metasurfaces (Figure S2).

**4.1 Quartz Sample Preparation and Cleaning**

The quartz substrates (Corning, 7980) were sequentially cleaned 10 mins in acetone, ethanol, and deionized water. After cleaning, residual liquids were blown off using N_2_ to ensure a clean, dry surface.

**4.2 Gold Thin Film Deposition and Dewetting**

A 5 nm-thick Au film was deposited on the silica surface via magnetron sputtering coater (MSP-300BT, CHUANG SHI WEI NA, Co., Ltd) with the process parameters of P=20 W; and N_2_ as inert gas with pressure of 20 Pa (Figure S3). The Au-coated samples were set in a furnace with a heating rate of 20°C/min up to 800°C, held for 30 min, and then cooled naturally inside the furnace. This process induced the formation of Au nanoislands due to solid-state dewetting. To realize the high-aspect-ratio Au nanoparticles, the sputtering/dewetting cycle was repeated three times (Figure S4).

**4.3 Reactive Ion Etching (RIE) for Nanostructuring**

The Au nanoislands served as an etching mask during RIE (reactive gas: CF_4_; pressure: 0.2 Pa; gas flow: 20 sccm; RF power: 40 W), enabling high-aspect-ratio nanostructuring of the silica substrate (Figure S5). The high etching selectivity between silica materilas and Au allowed high-aspect-ratio nanostructures.

**4.4 Surface Chemical Treatment for Superhydrophobicity**

The nanostructured silica substrates were immersed in a fluorosilane-ethanol solution (2% v/v) for 12 hours to form a hydrophobic self-assembled monolayer (SAM). Subsequently, the samples were cured in an oven for 2 hours to enhance surface stability and adhesion. The combined nanostructuring and fluorination approach converts the originally hydrophilic silica substrate into a superhydrophobic surface with high water contact angles (>150°) (Figure S5).

**Note S5.** Fabrication process and characterization of the nanostructures inside the silica glass. (Figure S16)

The femtosecond laser processing system comprises a femtosecond laser, an energy control module (comprising a half-wave plate (HWP) and a polarization beam splitter (PBS)), a polarization control module (consisting of a linear polarizer (LP), an electro-optical modulator (EOM), and a quarter-wave plate (QWP)), a real-time observation module (comprising light and a CCD camera), and a focused processing module (consisting of an objective lens and a 3D stage). The EOM is used to achieve real-time control of the polarization direction by applying different voltage values.

The femtosecond laser (wavelength: 1030 nm, repetition rate: 205 kHz, pulse duration: 450 fs, pulse energy: 1.1 μJ) employed in this study is generated by a Yb-doped potassium gadolinium tungstate (Yb:KGW)-based mode-locked regenerative amplified femtosecond laser system (PH2-20, Light Conversion Ltd.). An objective lens (10x, NA=0.26) is utilized to focus the femtosecond laser beam inside the silica glass. The nanostructures are fabricated via line-by-line and layer-by-layer laser direct writing by a computional controlled 3D stage. The line spacing is set at 1 μm, and the scanning speed is maintained at 6 mm/s. The layer spacing is 40 μm.

For the characterization of laser-induced nanostructures inside the silica glass, the sample undergoes polishing and subsequent etching in a 1 mol/L KOH solution for 24 hours. The prepared sample are observed by a scanning electron microscope (SEM, Thermo Scientific Helios 5 CX).

**Note S6.** Multi-factor influences in the environment.

The combined effects of multiple environmental factors are critical for advancing metasurface technologies. While our present study investigated individual influences under controlled conditions, real-world operation involves simultaneous exposure to sunlight, rain, dust, temperature fluctuations, wind, and complex electromagnetic environments. Such considerations are essential for applications ranging from passive radiative cooling, outdoor wireless communications (RIS for 5G/6G), and wireless power transfer to imaging, sensing, photovoltaics, beam steering, and environmental monitoring. Future work will focus on representative environments (urban, marine, desert, near-space) by designing simulators to accelerate long-term multi-factor testing, and on establishing a database of results as a community resource.


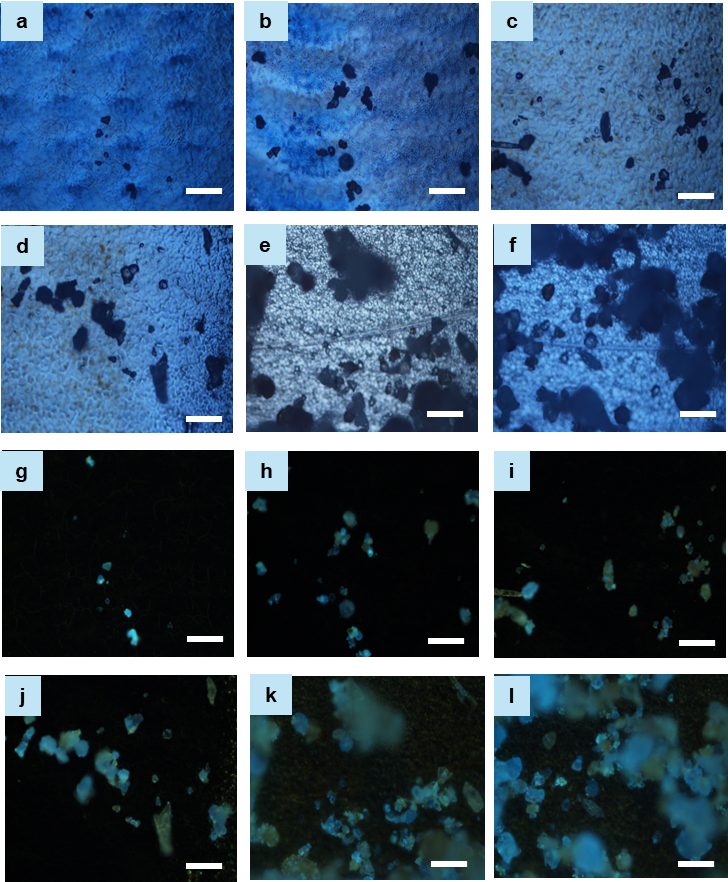


**Figure S1.** Different microscope images of the remaining particles on the laser textured surface corresponds to the homogeneous nano-textured surfaces A1 to A3 in the manuscript. An area of 870 × 653 µm taken by different area images are analyzed to calculate the particle size distribution. The statistical distribution counts the properties of the particles in all these images a) to f) are the wide-field microscope images and g) to l) are the fluorescent images to double-check the size distribution of the particles and exclude the defects. Scale bar: 50 μm.


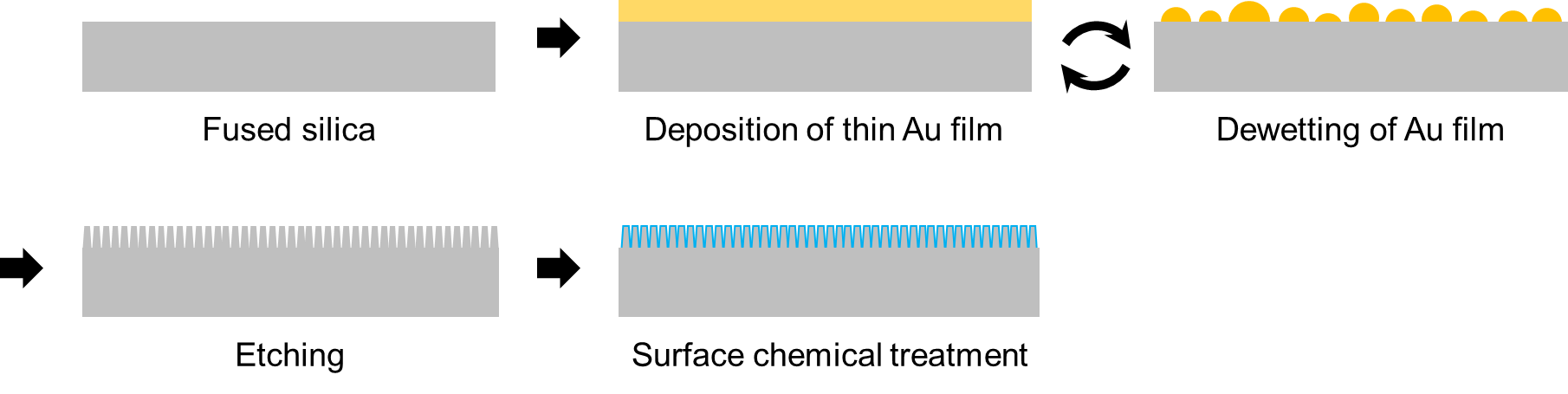


**Figure S2.** Fabrication process of the functional nanostructures of armored metasurfaces. The process includes the formation of nanomask on the surface of fused silica through the dewetting process of the gold film, the formation of a high-density nanopillar structure with a high aspect ratio on the fused silica surface through RIE, and the subsequent surface fluorination treatment to achieve a functional surface with high transmittance and waterproofing.

**Table S2.** Benchmarking with the parameters of surface texturing techniques in other literature.

| **Parameter** | **This work** | **Literature 1** | **Literature 2** | **Literature 3** | **Literature 4** | **Literature 5** | **Literature 6** | **Literature 7** | **Literature 8** | **Literature 9** | **Literature 10** | **Literature 11** | **Literature 12** |
| --- | --- | --- | --- | --- | --- | --- | --- | --- | --- | --- | --- | --- | --- |
| Anti-water (Contact Angle: °） | 158 | N.A. | 165 | 155.7 | 156 | N.A. | 156 | N.A. | 78 | N.A. | N.A. | 160 | 137.5 |
| Anti-icings (Freezing time: second） | 90 | N.A. | N.A. | N.A. | 1200 | 600 | N.A. | N.A. | N.A. | N.A. | N.A. | N.A. | N.A. |
| Anti-fogging | Yes | N.A. | N.A. | N.A. | N.A. | N.A. | N.A. | Yes | No | N.A. | No | Yes | N.A. |
| Anti-reflection (Reflectivity: %） | 1% | 0.45% | 0% | 5% | 2% | N.A. | N.A. | 32.90% | N.A. | 30% | 0.41% | 14% | 2% |
| Anti-impact (Pressure: kPa) | 795 | 1 | N.A. | N.A. | N.A. | N.A. | N.A. | N.A. | N.A. | N.A. | N.A. | N.A. | N.A. |
| Anti-scratch (Pressure: Mpa) | 50 | 2000 | N.A. | N.A. | N.A. | N.A. | N.A. | N.A. | N.A. | N.A. | N.A. | N.A. | N.A. |
| Heat-resistance (Temperature: ℃) | 1000 | 1000 | N.A. | N.A. | N.A. | N.A. | N.A. | N.A. | 1000 | 930 | 1000 | N.A. | N.A. |
| Anti-dust | Yes | N.A. | N.A. | N.A. | N.A. | N.A. | Yes | N.A. | N.A. | N.A. | N.A. | N.A. | N.A. |

| Pulication | Reference |
| --- | --- |
| Literature 1 | Optica, 2020, 7, 518 |
| Literature 2 | Applied Acoustics, 2021, 180, 108139 |
| Literature 3 | Applied Surface Science. 2019, 463, 741 |
| Literature 4 | Iscience, 2024, 27, 111086. |
| Literature 5 | Nature Communications, 2023, 14, 8096 |
| Literature 6 | Nano Energy, 2023, 114, 108625 |
| Literature 7 | Nature Nanotechnology, 2023, 18, 137. |
| Literature 8 | Advanced Science, 2023, 10, 2301111 |
| Literature 9 | Laser Photonics Rev. 2024, 18, 2400007 |
| Literature 11 | ACS Nano, 2020, 14, 11712 |
| Literature 12 | Scientific Reports, 2025, 15, 6322 |


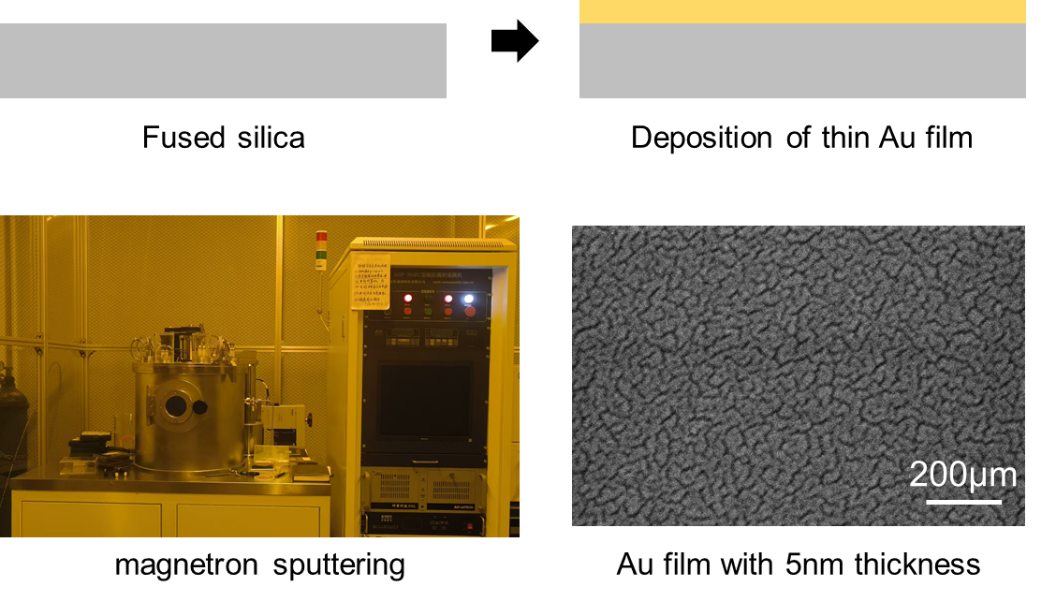


**Figure S3.** Magnetron sputtering of Au film with 5nm thickness. The thickness of the gold film coated on the silica surface is 5nm each time, and the microcracks on the surface are helpful to form island-like Au nanoparticles in the subsequent high temperature treatment process.


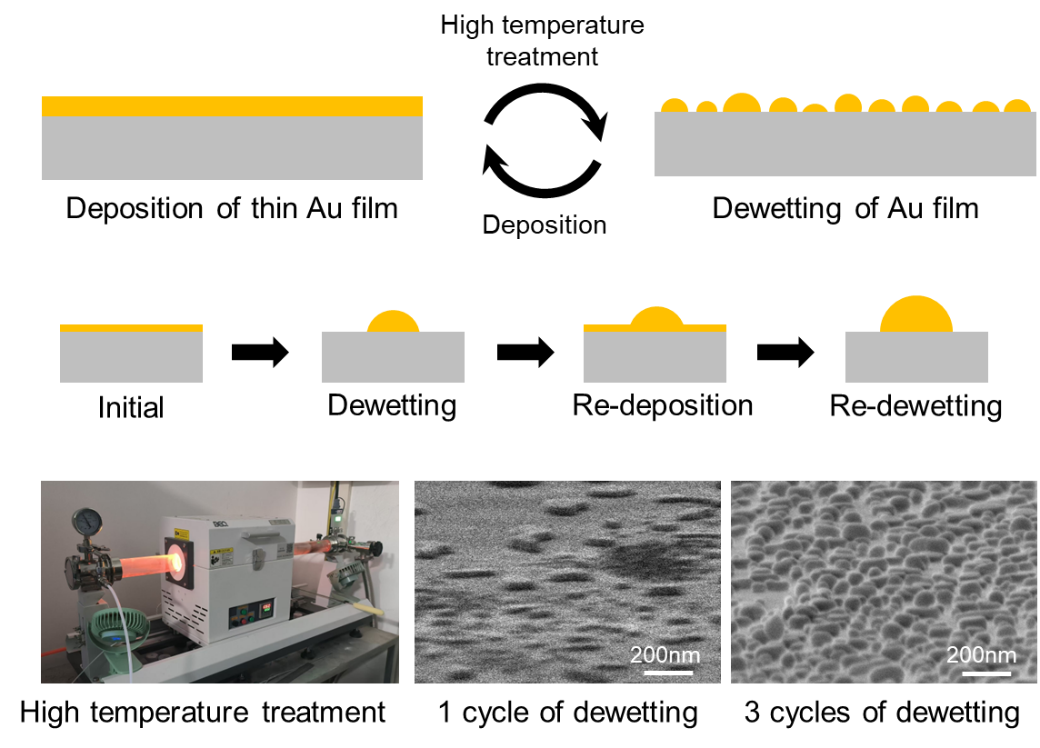


**Figure S4.** Formation of Au nanoparticles with high aspect ratio on the silica substrate. Gold film was transformed into a gold nanoislands after high temperature treatment, and the density and aspect ratio of the islands were increased by repeated deposition-annealing treatment.


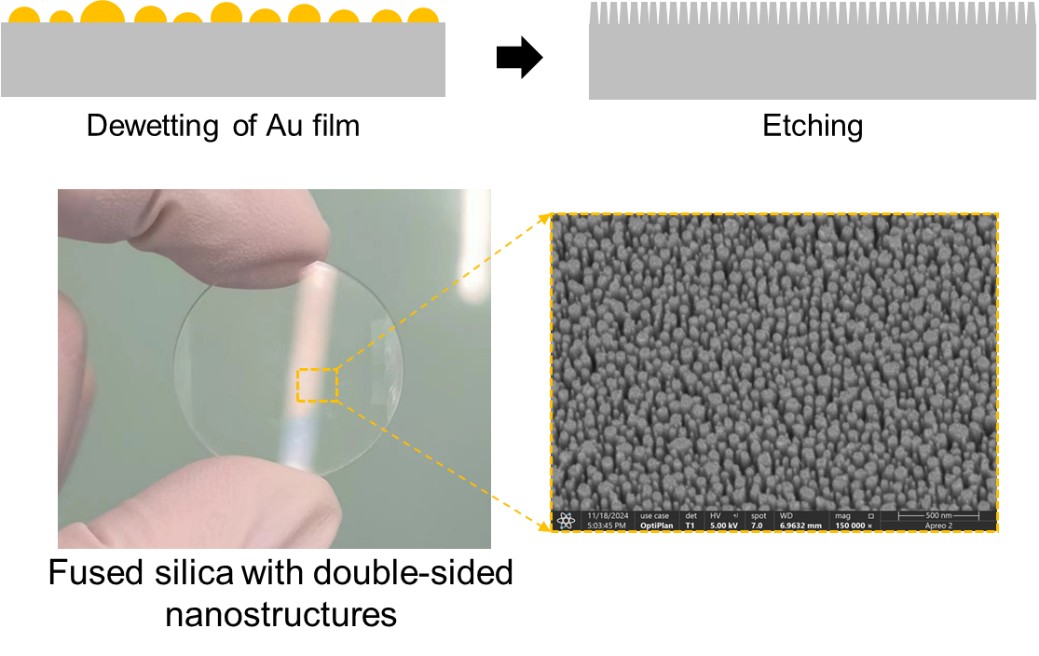


**Figure S5.** Preparation of nanostructures on the silica surface by reactive ion etching (RIE). The formed nanopillars/nanocones range in size from 10-200 nm at the top and about 600 nm in height.


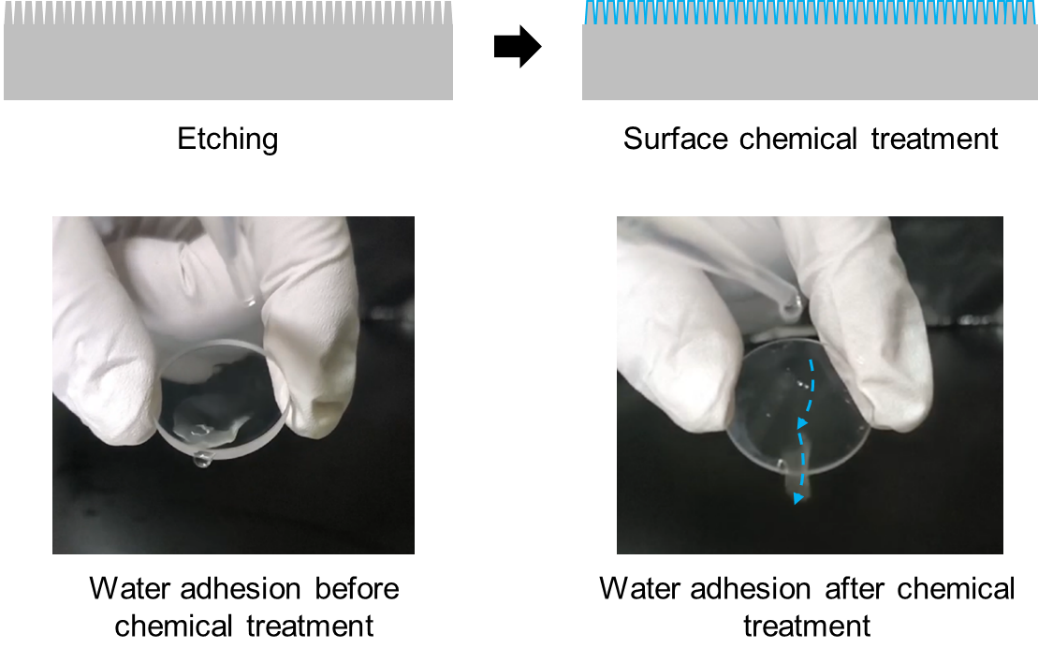


**Figure S6.** Surface fluorosilane treatment for the preparation of superhydrophobic nanostructures.


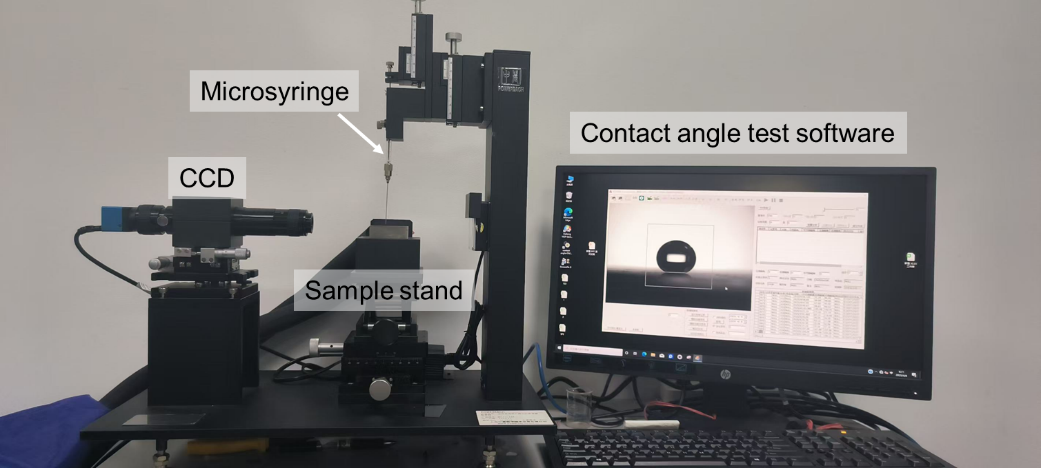


**Figure S7.** The contact angle measuring instrument is used to test the wettability of liquid on the surface of the sample. The wettability of droplets on the surface of the sample was characterized by contact angle. The 8uL deionized water was dropped on the sample surface, and then the contact state of the droplet on the sample surface was recorded by CCD, and then the contact angle of the droplet on the sample surface was measured by the contact angle measurement software.


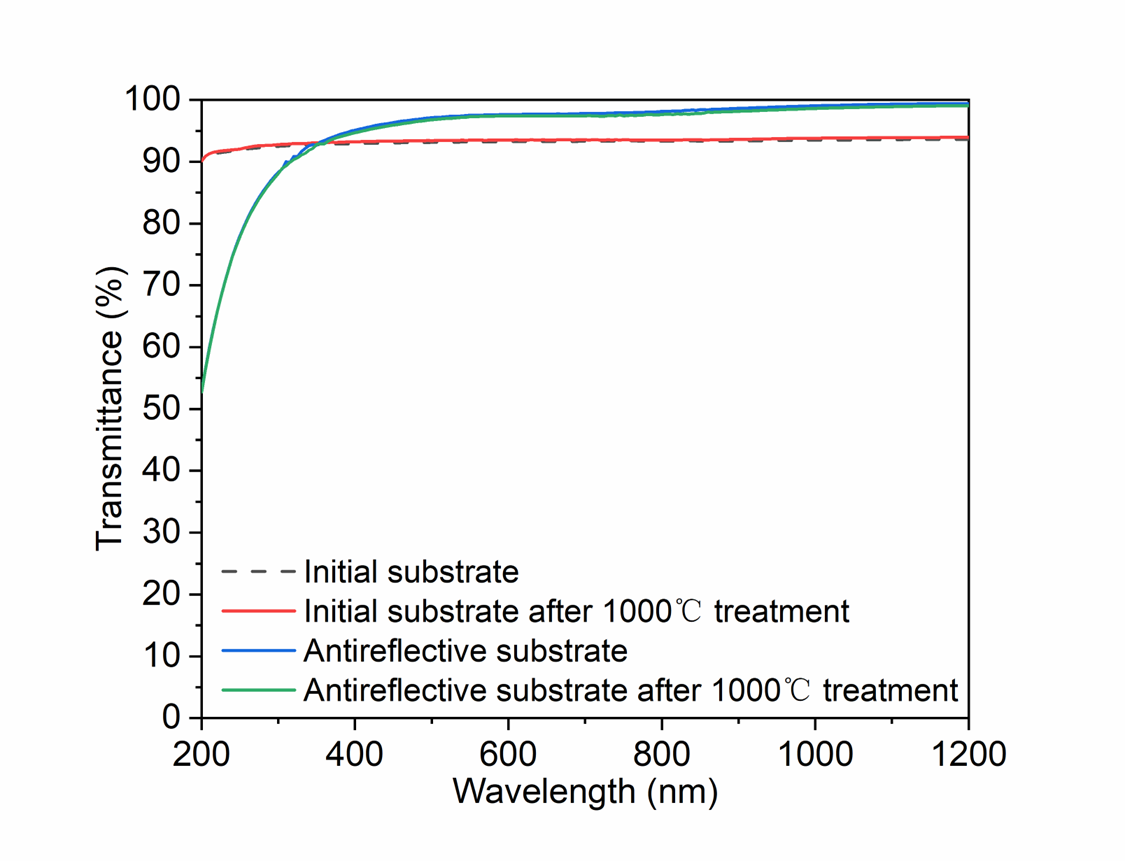


**Figure S8.** High-temperature stability of the metasurface. The metasurface was subjected to a thermal shock at 1000℃ for 30 minutes to test the thermal endurance. Pre- and post-treatment transmittance characterization revealed that the nanostructures remained consistent with no evidence of melting or structural degradation.


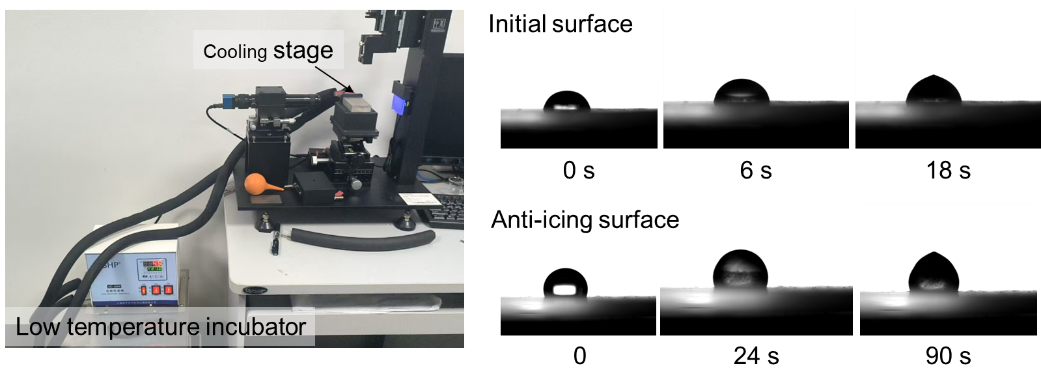


**Figure S9.** Anti-icing performance of metasurface. The anti-icing performance of the metasurface is characterized by a contact angle measuring instrument, and the cold table temperature is controlled at-20 °C by a low-temperature incubator. The sample was placed on a cold table, and 8 μL of droplets were injected on the surface of the sample through a microsyringe, and then record the solidification process of the droplets.


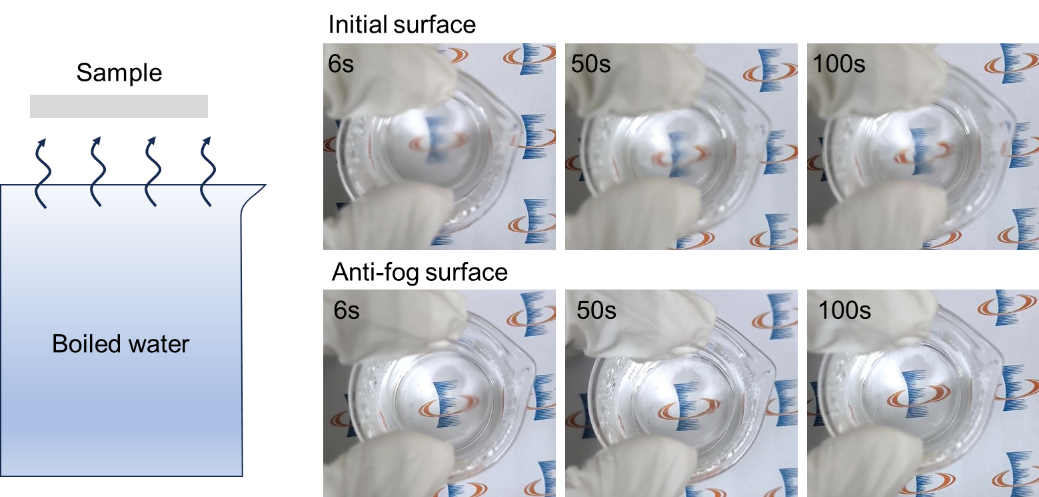


**Figure S10.** Antifouling performance of metasurface. The deionized water was poured into the beaker, and then heated to boiling through the heating table. The metasurfaces without surface chemical treatment and surface hydrophobic treatment were placed above the beaker, respectively. The surface fog condensation process was recorded in real time by the camera, and the surface transmittance of the two metasurfaces was compared within 100s.


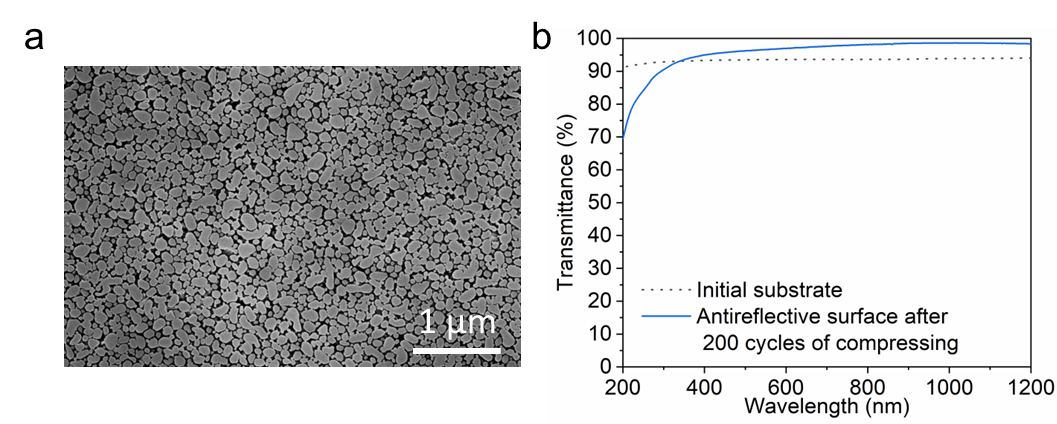


**Figure S11.** Characterization of the robustness of the antireflective nanostructures after 200 cycles of compressing. (a) SEM image of the nanostructures after 200 cycles of compressing. (b) Optical characterization of the nanostructures after 200 cycles of compressing.


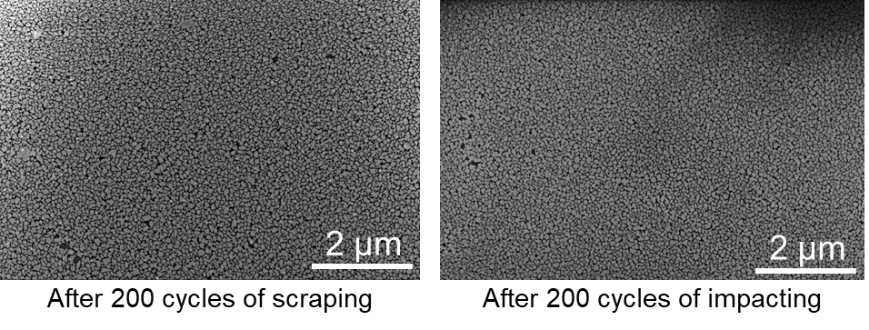


**Figure S12.** SEM images of the antireflective structures after 200 cycles of scraping and impacting.


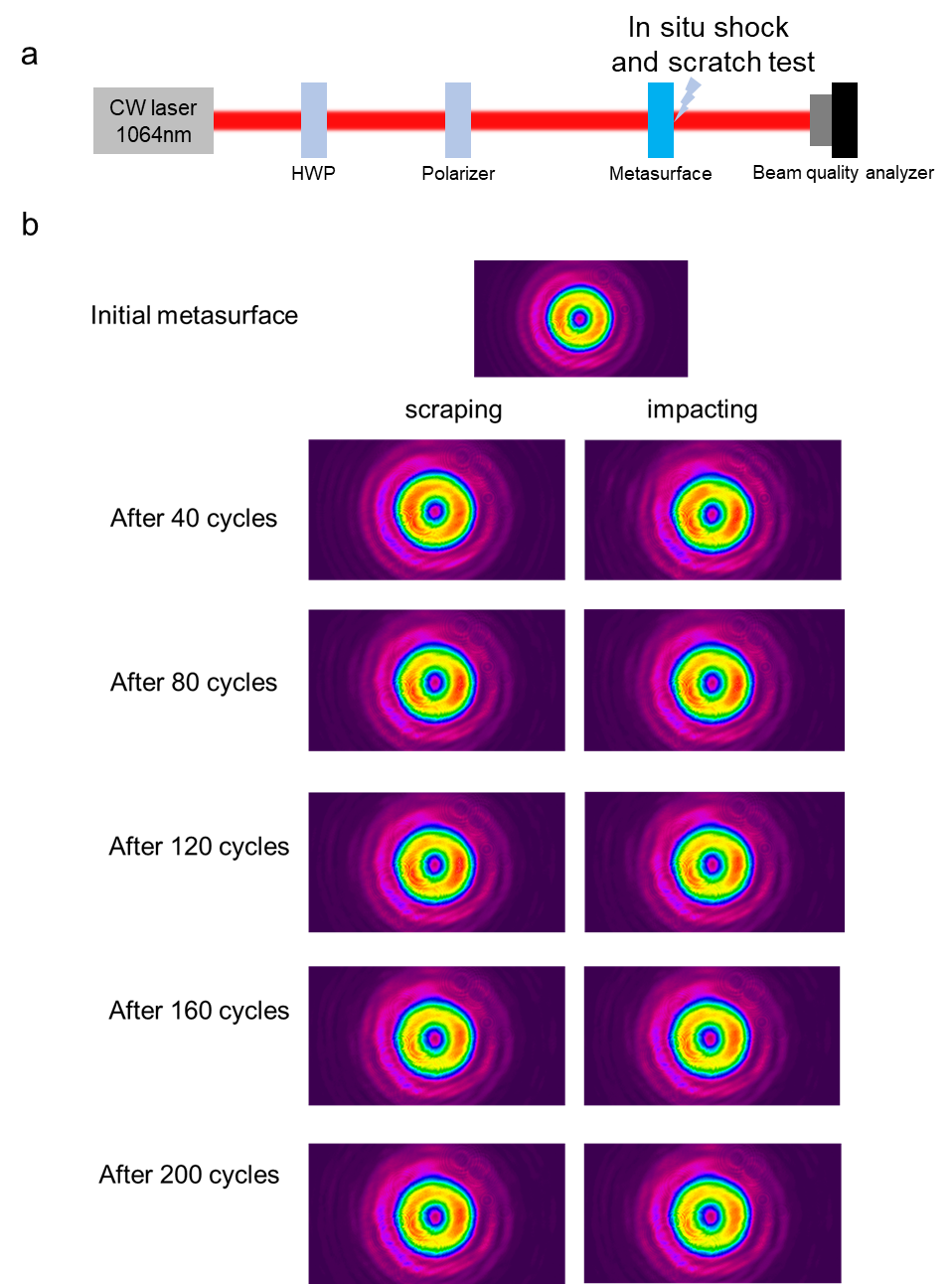


**Figure S13.** Test on the quality of vortex beam after cycles of surface scraping and impacting. (a) Vortex beam quality in situ testing system. (b) Measured vortex beam after surface treatment.

.



**Figure S14.** Measured transmittance as a function of wavelength across several angles of incidence.


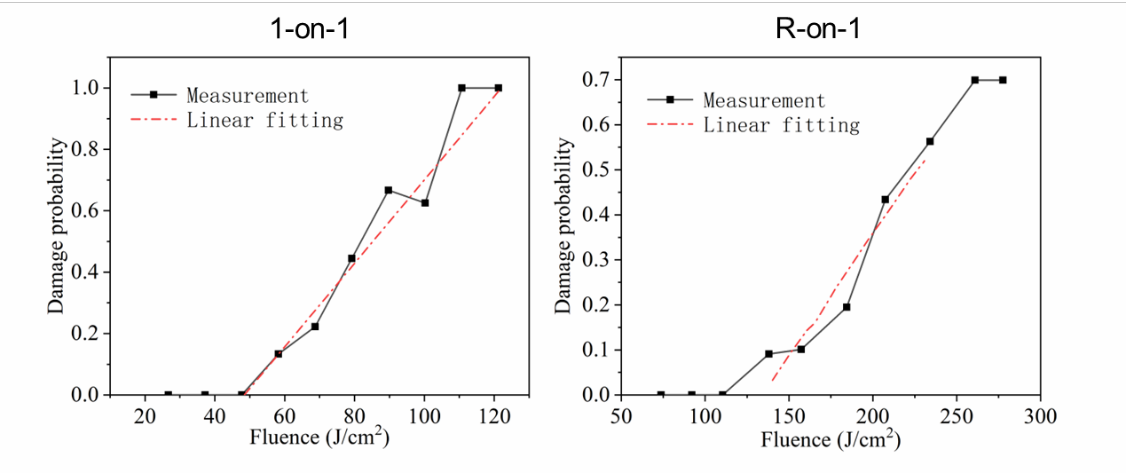


**Figure S15.** Laser induced damage threshold of the prepared metasurface. To showcase the resistance of the prepared metasurfaces against intense laser exposure, we assessed the LIDTs using the standardized 1-on-1 and R-on-1 method outlined in ISO 21254. The testing apparatus utilized a laser with a wavelength of 1064 nm and a pulse duration of 6 ns.


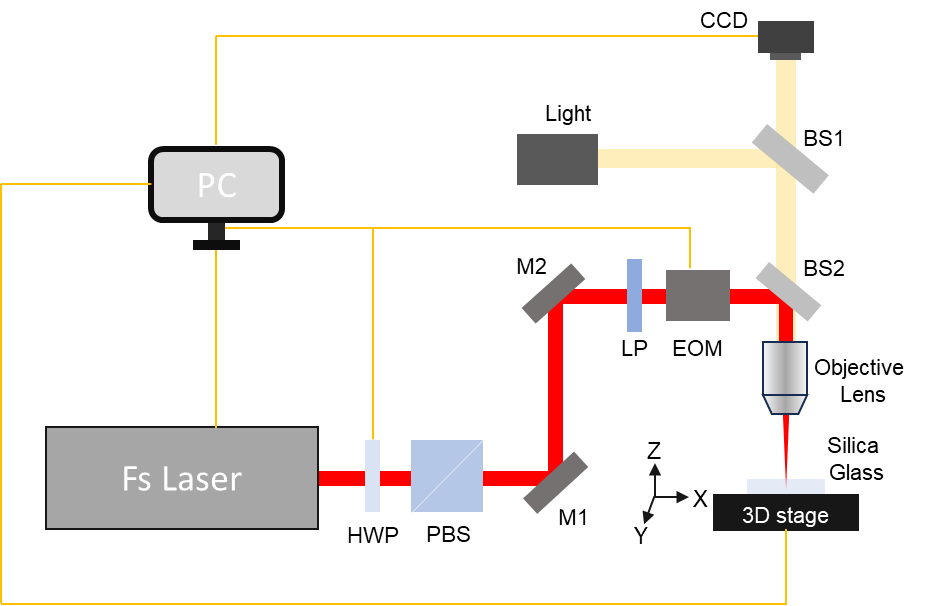


**Figure S16.** Schematic diagram of the femtosecond laser processing system.

**Table S3.** Metasurface Applications and Functions.

The field of metasurfaces has advanced rapidly in recent years, with applications expanding from consumer electronics to healthcare and defense. Commercial products already integrate meta-optics at large scale, with more than 140 million units deployed in smartphones and tablets, primarily enabled by companies such as Metalenz, in collaboration with UMC for volume manufacturing, and major OEMs in Asia. Current applications include compact lenses, depth sensors, face recognition modules, polarization-based imaging for biometrics and material identification, 3D sensing and LiDAR, lightweight optics for AR/VR, miniaturized medical imaging devices, and compact defense-oriented optical systems as summarized in the Table S3. While these demonstrate multifunctionality, integration, and advanced polarization control, the surface proposed in this work remains at an early stage, with more limited functionalities. Nevertheless, its development has the potential to complement existing approaches by improving reproducibility, reducing fabrication defects, and lowering production costs. To provide context, we include an additional discussion in the main text and a supplementary section summarizing recent advances and commercial progress in metasurfaces, highlighting their growing impact in addressing large-scale consumer and industrial demands.

| Application Area | Examples & Functions | Commercial Status / Notes |
| --- | --- | --- |
| Consumer Electronics (Smartphones / Tablets) | Compact lenses, depth sensors, dot projectors, face recognition modules | Already mass-shipped in phones (Metalenz: >140 million units) |
| Biometrics & Security | Polarization-based face ID (e.g., *Polar ID*), 3D face authentication, anti-spoofing | Emerging as full-stack systems, not just optics |
| 3D Sensing & Imaging | Depth mapping, structured-light projectors, LiDAR modules | Used in mobile devices, expanding to automotive and robotics |
| Polarization Imaging | Captures polarization contrast for material ID, medical imaging, anti-counterfeiting | Commercial prototypes available |
| Augmented / Virtual Reality (AR/VR) | Lightweight optics, waveguide couplers, holographic displays | Early-stage, under R&D and pilot production |
| Automotive & Mobility | LiDAR beam shaping, driver monitoring, in-cabin sensing | In testing, not yet widely deployed |
| Medical & Health | Miniaturized endoscopes, NIR imaging, biosensing (e.g., detecting pathogens, glucose monitoring) | Mostly pre-commercial but with strong research-industry interest |
| Industrial & Defense | Compact imaging systems, optical communications, IR/thermal sensing | Pilot or defense-specific projects |

**Movie S1.** Hologram Characterization of Armored Metasurface Before/After Mechanical Impact Testing.


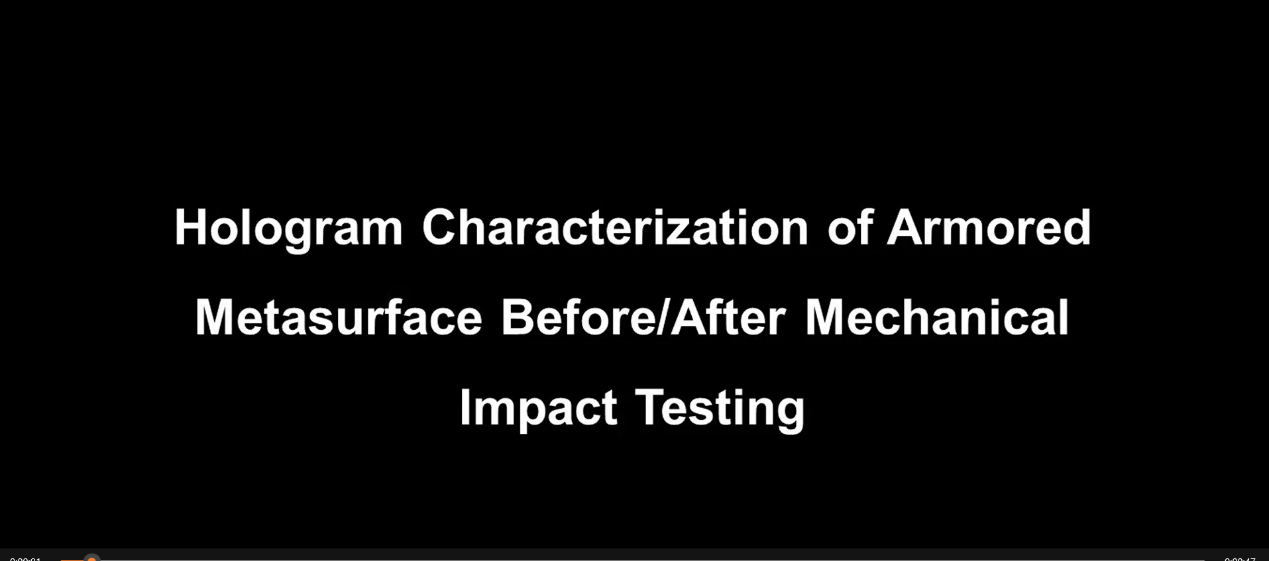

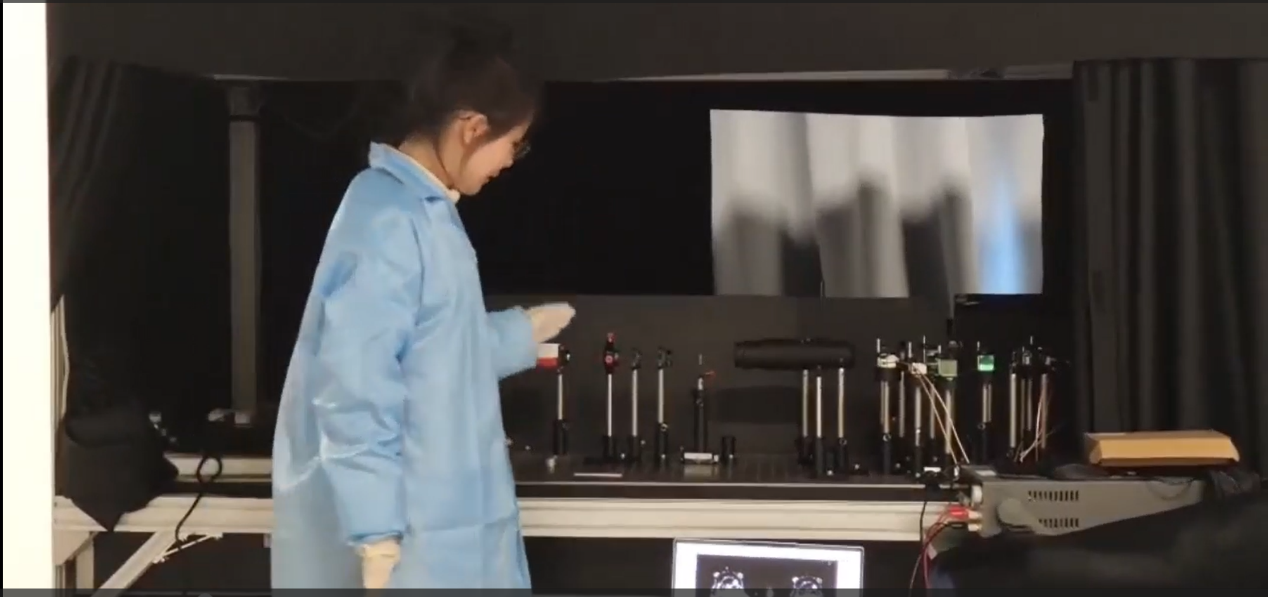


**Movie S2.** Anti-Turbulence Performance of the Armored Metasurfaces


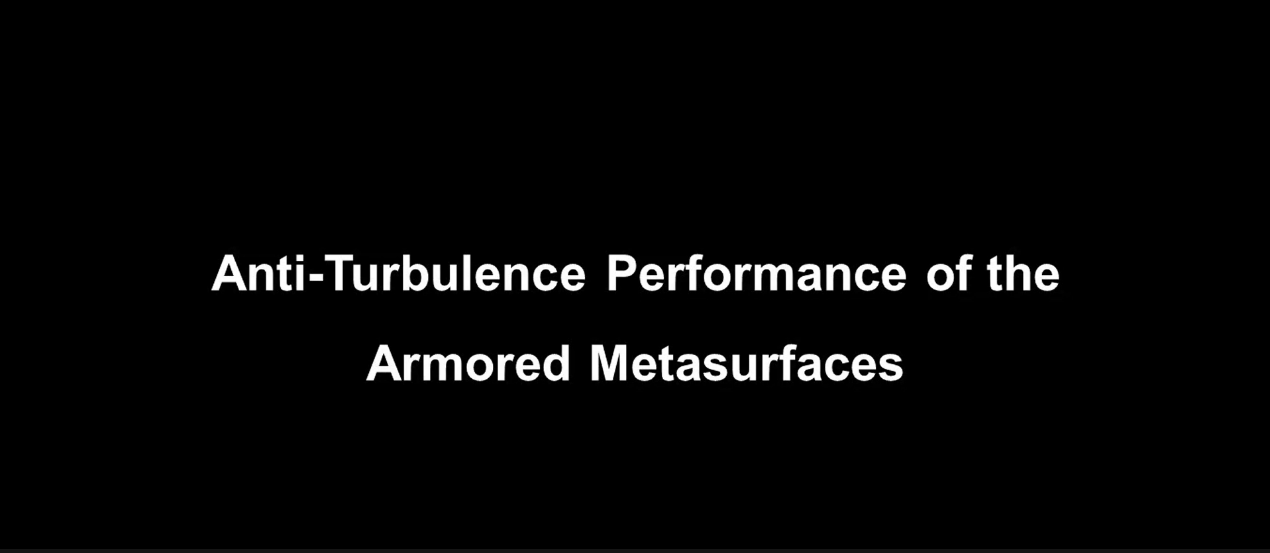

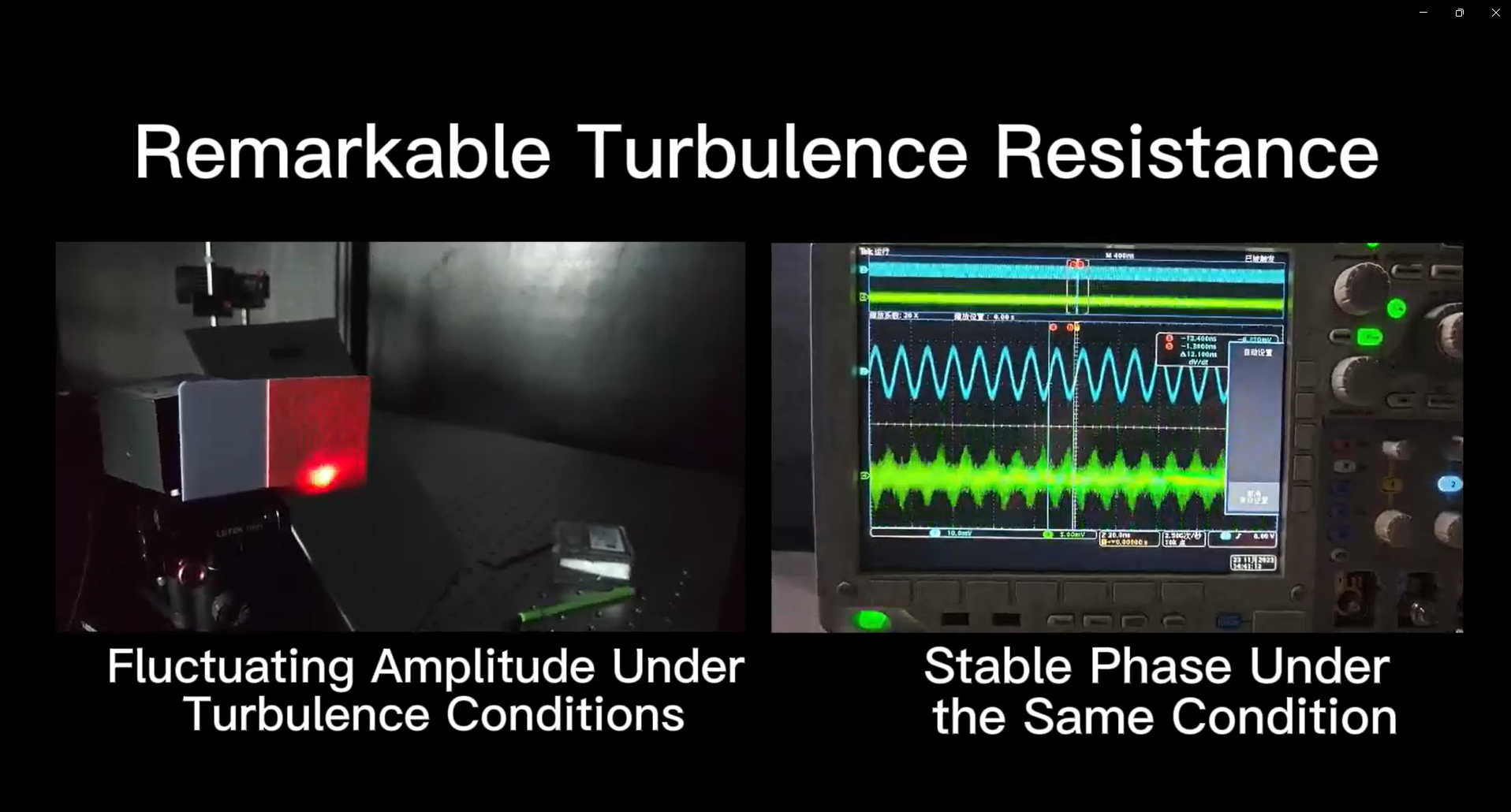

Supplement: Supplementary file 1 — Supporting Information [file ADVS-13-e14000-s001.docx]
